# Supplementary material for: Layering genetic circuits to build a single cell, bacterial half adder
Source: BMC Biol. 2015 Jun 16;13:40. doi: 10.1186/s12915-015-0146-0 (PMC4490610; doi:10.1186/s12915-015-0146-0)
Supplement: Additional file 1: — Supplementary methods, figures and tables. [file 12915_2015_146_MOESM1_ESM.docx]

**CONTENT PAGE PAGE NO.**

Predicting the Effect of RBS on Transfer Function of Input Devices...……………. 3

Supplementary Table 1. Transfer functions of input switch devices derived from experimental results and model prediction….......................................................... 4

Supplementary Fig. 1. Predicting the transfer functions of input devices with different ribosome binding sites……………………………………………………………. 5

Supplementary Fig. 2**.** Design of input device with strong and weak ribosome binding sites……………………………………………………………………………........ 6

Supplementary Fig. 3. Design of input device B with strong and weak ribosome binding sites………………………………………………………………………... 7

Supplementary Fig. 4. Pairing compatibility assessment of (A) pRHAB promoter with RFP expression, and (B) pBAD promoter with GFP expression…………...... 8

Supplementary Fig. 5. Effects of genetic architecture on the switching behaviour of σ54-dependent pHrpL promoter.................................................................... 9 – 10

Supplementary Fig. 6. Directed evolution and characterisation of lambda repressor binding sites................................................................................................... 11

Supplementary Fig. 7. Flow cytometry analysis of the biological half adder on a 2D contour plot.................................................................................................... 12

Supplementary Fig. 8. The effect of plasmid copy number in the shunting and sequestering of transcription factors.............................................................. 13

Supplementary Fig. 9. Cell viability profile of the biological half adder after 4 hours of induction......................................................................................................... 14

Predicting the Effect of RBS on Transfer Function of IMPLY Gates............... 15

Supplementary Table 2. List of parameters used in the modelling of IMPLY logic gates................................................................................................................ 15

Supplementary Fig. 10: Predicted transfer functions of the IMPLY gate with different ribosome binding sites at steady state............................................................. 16

Model-aided Design of AND, OR & XOR Logic Gates................................... 17 – 22

Supplementary Table 3. Transfer functions of input switch devices in the modelling of AND and OR logic gate.................................................................................. 18

Supplementary Fig. 11**.** Predicted normalised output of HrpRS AND gate..... 19

Supplementary Fig. 12**.** Predicted normalised output of pBAD-pRHAB OR gate................................................................................................................. 21

Supplementary Fig. 13. Predicted normalised outputs of 4 different OR gate designs…………………………………………………………………………….... 22

Sequence Design……………………………………………………………………….... 24

References.............................................................................................................. 47

**Predicting the Effect of RBS on Transfer Function of Input Devices**

To enable model-driven design and optimization of synthetic biological systems with more complicated circuitry and computational functions, we examine the effect of ribosome binding sites (RBS) on the steady state transfer function of input switch devices. By analysing reference data [[1](#_ENREF_1)] that had previously characterise the input-output relationship of genetic switches in the form

$$Output Y=A+ \frac{B\left( X^{n} \right)}{C^{n}+ X^{n}} \mathbf{Eqn.}(1)$$

Where A, B, C and n are empirically derived parameter and X is input concentration,

, we observed that parameters that are most sensitive to changes in RBS are parameters A and B. Accordingly, knowing the relative output of switch devices with weaker RBS by either prediction from reliable software or by single experimental measurement of device’s output at input maximal, the parameters A and B can be scaled proportionally to obtain *a priori* parameters that accurately describe the transfer function of other devices with weaker RBS (Supplementary Fig. 1A). We validated our approach with previously obtained data sets (Supplementary Table 1) and showed that the transfer function of input devices pLuxR (Supplementary Fig. 1B) and pBAD (Supplementary Fig. 1C) with different RBS can be reliably estimated without excessive experimentation.

**Supplementary Table 1.** Transfer functions of input switch devices derived from experimental results and model prediction. Input devices marked with * were used to derive the values of parameter A and B for other devices with the same inducible promoter but with different RBS.

| **Input Device** | **A** | **B** | **C (mM)** | **n** | **R^2^** | **Ref** |
| --- | --- | --- | --- | --- | --- | --- |
| pLuxR-Rbs34-GFP* | 2010.01 | 1.349E+05 | 2.890E-09 | 1.898 | 0.994 | [[1](#_ENREF_1)] |
| pLuxR-Rbs31-GFP | 969.32 | 7.693E+04 | 2.955E-09 | 1.771 | 0.999 | [[1](#_ENREF_1)] |
| pLuxR-Rbs32-GFP | 581.16 | 5.143E+04 | 3.509E-09 | 1.655 | 0.999 | [[1](#_ENREF_1)] |
| pLuxR-RbsH-GFP | 364.12 | 3.194E+04 | 3.784E-09 | 1.616 | 0.998 | [[1](#_ENREF_1)] |
| pLuxR-Rbs34-GFP | 2010.01 | 1.349E+05 | 2.890E-09 | 1.898 | 0.994 | This work |
| pLuxR-Rbs31-GFP | 1198.15 | 7.693E+04 | 2.890E-09 | 1.898 | 0.986 | This work |
| pLuxR-Rbs32-GFP | 801.00 | 5.143E+04 | 2.890E-09 | 1.898 | 0.965 | This work |
| pLuxR-RbsH-GFP | 497.45 | 3.194E+04 | 2.890E-09 | 1.898 | 0.946 | This work |
|  |  |  |  |  |  |  |
| pBAD-Rbs34-GFP* | 705.50 | 1.411E+05 | 5.240E-04 | 1.173 | 0.999 | [[1](#_ENREF_1)] |
| pBAD-RbsH-GFP | 304.56 | 9.229E+05 | 4.800E-04 | 1.415 | 0.999 | [[1](#_ENREF_1)] |
| pBAD-Rbs32-GFP | 135.41 | 5.208E+04 | 5.160E-04 | 1.268 | 0.999 | [[1](#_ENREF_1)] |
| pBAD-Rbs33-GFP | 16.77 | 1.290E+04 | 5.130E-04 | 1.323 | 0.999 | [[1](#_ENREF_1)] |
| pBAD-Rbs34-GFP | 705.50 | 1.411E+05 | 5.240E-04 | 1.173 | 0.999 | This work |
| pBAD-RbsH-GFP | 461.45 | 9.229E+05 | 5.240E-04 | 1.173 | 0.947 | This work |
| pBAD-Rbs32-GFP | 260.40 | 5.208E+04 | 5.240E-04 | 1.173 | 0.979 | This work |
| pBAD-Rbs33-GFP | 64.50 | 1.290E+04 | 5.240E-04 | 1.173 | 0.983 | This work |

**Supplementary Fig. 1.** Predicting the transfer functions of input devices with different ribosome binding sites (RBS). Validation of the suggested forward engineering approach with (**A**) pLuxR and (**B**) pBAD expression systems with different ribosome bind sites upstream of GFP reporter. Solid lines represent the forward predicted transfer functions, with corresponding R-squared values, while symbols represent empirically-derived transfer functions using total experimental fitting. All the empirically-derived transfer functions have R-squared values of 0.998 or greater and are obtained from an earlier study by Wang et al 2011.

**Supplementary Fig. 2.** Design of input device A with **(A)** strong and **(B)** weak ribosome binding sites, respectively. Input device A is modelled after the arabinose-induced expression system. In the presence of arabinose, AraC binds and triggers conformational change in the pBAD promoter to activate RFP expression. The RhaS transcription factor (for use with rhamnose-inducible expression system) is included in the design to enable accurate characterisation of input device B in the context of the overall half adder genetic circuit. **(C)** Transfer function of the pBAD promoter. Green triangles represent experimental data, while black line represents empirically-derived transfer function for construct with a strong ribosome binding site - as denoted in the equation above. The blue line represents the predicted transfer function for a construct with a weak ribosome binding site. Error bars represent the standard deviation of biological triplicates.

**Supplementary Fig. 3.** Design of input device B with **(A)** strong and **(B)** weak ribosome binding sites, respectively. Input device B is modelled after the rhamnose-induced expression system. In the presence of rhamnose, RhaS binds and triggers conformational change in the pRHAB promoter to activate RFP expression. The AraC transcription factor (for use with arabinose-inducible expression system) is included in the design to enable accurate characterisation of input device B, in the context of the overall half adder genetic circuit. **(C)** Transfer function of pRHAB promoter. Blue diamonds represent experimental data, while black line represents an empirically-derived transfer function for a construct with strong ribosome binding site, as denoted in the equation above. The red line represents the predicted transfer function for construct with weak ribosome site. Error bars represent the standard deviation of biological triplicates.

**Supplementary Fig. 4.** Pairing compatibility assessment of **(A)** pRHAB promoter with RFP expression, and **(B)** pBAD promoter with GFP expression. The red squares and green triangles show the characteristics of each promoter following induction with its natural and heterologous sugar, respectively. The blue diamonds represent the performance of each input device in the presence of both sugars. Error bars represent standard deviation of biological triplicates.

**Supplementary Fig. 5.** Effects of genetic architecture on the switching behaviour of σ54-dependent pHrpL promoter. **(A)** pHrpL-GFP reporter in high copy plasmid, **(B)** pHrpL-GFP reporter assembled upstream of pHrpL-λCl in high copy plasmid, **(C)** pHrpL-GFP reporter assembled downstream of pHrpL-λCl in high copy plasmid, **(D)** pHrpL-GFP reporter assembled downstream of λCl in high copy plasmid, **(E)** pHrpL-GFP reporter and pHrpL-λCl separately assembled in low and high copy plasmids, respectively, and **(F)** pHrpL-GFP reporter assembled in both low and high copy plasmids. Within the same plasmid vector, an pHrpL promoter placed upstream is able to turn on a proximate, downstream pHrpL promoter and caused GFP expression - even in the absence of HrpRS transcription activators. This genetic context dependent effect can be circumvented by insulating pHrpL expression cassettes in different plasmids. **(G)** Quantitative characterisation of pHrpL promoter leakiness in different genetic contexts. Error bars represent the standard deviation of three independent experiments.

**Supplementary Fig. 6.** Directed evolution and characterisation of lambda repressor binding sites. **(A)** Layout of genetic circuit used in the mutation and screening of lambda repressor binding sites. Characteristic DNA sequences of the lambda repressor binding sites with perfect dyad symmetry were mutated and screened for RFP expression in the presence of arabinose (input A) and suppression of RFP in the presence of both arabinose and rhamnose (input B). Lambda repressor binding sites located downstream of pBAD promoter transcription start site (denoted with +1) are denoted as red and green letters (reverse complement). Key mutations that generated the best repressor in this screen are marked by green boxes. **(B)** Characterisation of original (blue bars) and evolved lambda repressor binding sites (red bars) in the engineered genetic circuit. Error bars represent the standard deviation of biological triplicate.

**Supplementary Fig. 7.** Flow cytometry analysis of the biological half adder on a 2D contour plot. (A) Half adder without any inputs, (B) half adder with only arabinose (input A), (C) half adder with only rhamnose (input B) and (D) half adder with both inputs A and B. FITC-A and PE-CF594-A represent channels that detect GFP and RFP fluorescence, respectively.

**Supplementary Fig. 8.** The effect of plasmid copy number in the shunting and sequestering of transcription factors. GFP output of the pure AND gate is higher than the GFP output of half adder’s AND gate. Error bars represent the standard deviations for four independent experiments.

**Supplementary Fig. 9.** Cell viability profile of the biological half adder after 4 hours of induction. Error bars represent the standard deviations for four independent experiments.

**Predicting the Effect of RBS on Transfer Function of NIMPLY Gates**

We characterised the steady state transfer function of the NIMPLY gate with strong RBS by titrating cells that were activated for RFP expression by a fixed input of B above switch point with variable concentration of input A (Fig. 4C). Our result shows that the NIMPLY gate can be described mathematically by a Hill-like equation of the form

$$IMPLY=A+ \frac{\alpha B\left( C^{n} \right)}{C^{n}+ X^{n}} \mathbf{Eqn.}\left( 2 \right)$$

$$IMPLY=571+ \frac{2278\alpha\left( {5.66 \times{10}^{-05})}^{0.718} \right)}{{(5.66 \times{10}^{-05})}^{0.718}+ {[Ara]}^{0.718}} \mathbf{Eqn.}\left( 3 \right)$$

Where A, B, C and n are empirically derived parameter, X is input A concentration, and α = 1 in presence and 0 in absence of input B above switch point.

Drawing insights from earlier results, we hypothesized that parameters A and B are parameters that are most sensitive to the overall behaviour of NIMPLY gates when the RBS that initiate the expression of the RFP reporter are changed. To test our hypothesis, we simulated the output of NIMPLY gates whereby the RFP expressions are initiated from RBS of different strengths (modelling parameters as listed in Supplementary Table 2). The modelling results (Supplementary Fig. 10) are coherent with observed experimental results and show that RBS can be used for fine tuning of layered genetic circuits.

**Supplementary Table S2.** List of parameters used in the modelling of NIMPLY logic gates. NIMPLY circuit marked with * was empirically fitted using least square regression fitting (R^2^ = 0.998), and used to derive the values of parameter A and B for other NIMPLY circuits in order of decreasing output.

| **IMPLY GENETIC CIRCUIT** | **A** | **B** | **C (M)** | **n** |
| --- | --- | --- | --- | --- |
| pBAD-Rbs34-λCl-T-pBAD-pRHAB-RbsA1-RFP-T* | 571 | 2278 | 5.66E-05 | 0.718 |
| pBAD-Rbs34-λCl-T-pBAD-pRHAB-RbsA2-RFP-T | 420 | 1708 | 5.66E-05 | 0.718 |
| pBAD-Rbs34-λCl-T-pBAD-pRHAB-RbsA3-RFP-T | 270 | 1139 | 5.66E-05 | 0.718 |
| pBAD-Rbs34-λCl-T-pBAD-pRHAB-RbsB-RFP-T | 120 | 570 | 5.66E-05 | 0.718 |

**Supplementary Fig. 10**: Predicted transfer functions of the NIMPLY gate with different ribosome binding sites at steady state. Transfer function of the NIMPLY gate with the strongest ribosome binding site RbsA1 was obtained by empirical fitting.

**Model-aided Design of AND, OR & XOR Logic Gates**

To model an AND logic gate, transfer function of individual switch devices were incorporated to the following equation similar to how the effective resistance of a parallel arrangement of resistors was calculated.

$$\mathrm{AND}_{\mathrm{Abs}} = \left[ \frac{\alpha_{\mathrm{AND}}}{\frac{1}{TF1}+ \frac{1}{TF2}} \right] \mathbf{Eqn.} (4)$$

Where α_AND_ is an empirically-derived parameter, and TF1 and TF2 are transfer functions of input switch device 1 and 2 respectively.

This modelling approach is based on the assumption that at steady state, intracellular genetic expression occurs on a faster time scale than diffusion of inducers into the cells. Thus, the key factor which influences the characteristic and performance of genetic logic gates follows the transfer function of input genetic switches.

From Eqn. (1), the transfer functions of input 1 and 2 are presented as,

$$TF1=A_{1}+ \frac{B_{1}\left( {X_{1}}^{n1} \right)}{{C_{1}}^{n1}+ {X_{1}}^{n1}} \mathbf{Eqn.} (5)$$

$$TF2=A_{2}+ \frac{B_{2}\left( {X_{2}}^{n2} \right)}{{C_{2}}^{n2}+ {X_{2}}^{n2}} \mathbf{Eqn.} (6)$$

Substituting, Eqn. (5) and (6) into Eqn. (4), the normalised output of AND logic gate:

$$\mathrm{AND}_{\mathrm{Norm}}= \frac{\left[ \frac{\alpha_{\mathrm{AND}}}{\frac{1}{TF1}+ \frac{1}{TF2}} \right]}{\lim_{X_{1}X_{2}\to\infty} \left[ \frac{\alpha_{\mathrm{AND}}}{\frac{1}{TF1}+ \frac{1}{TF2}} \right]}$$

$$= \frac{\left[ \frac{1}{\frac{1}{A_{1}+ \frac{B_{1}\left( {X_{1}}^{n1} \right)}{{C_{1}}^{n1}+ {X_{1}}^{n1}}}+ \frac{1}{A_{2}+ \frac{B_{2}\left( {X_{2}}^{n2} \right)}{{C_{2}}^{n2}+ {X_{2}}^{n2}}}} \right]}{\lim_{X_{1}X_{2}\to\infty} \left[ \frac{1}{\frac{1}{A_{1}+ \frac{B_{1}\left( {X_{1}}^{n1} \right)}{{C_{1}}^{n1}+ {X_{1}}^{n1}}}+ \frac{1}{A_{2}+ \frac{B_{2}\left( {X_{2}}^{n2} \right)}{{C_{2}}^{n2}+ {X_{2}}^{n2}}}} \right]} \mathbf{Eqn.} (6)$$

From Eqn. (5) and (6), as X_1_ and X_2_ approaches infinity, TF_1_ and TF_2_ are B_1_ and B_2_ respectively. Thus, the normalised output of AND logic gate is derived as:

$$\mathrm{AND}_{\mathrm{Norm}} =\left[ \frac{B_{1}{+ B}_{2}}{B_{1}B_{2} \left( \frac{1}{TF1}+ \frac{1}{TF2} \right)} \right] \mathbf{Eqn.}(7)$$

**Supplementary Table 3.** Transfer functions of input switch devices in the modelling of AND and OR logic gates.

| **Input Device** (Rbs) | **A** | **B** | **C** | **n** | **Used in Modelling** |
| --- | --- | --- | --- | --- | --- |
| pBAD-(strong RBS) | 15 | 3885 | 1.16E-4 | 1.983 | OR GATE |
| pBAD-(weak RBS) | 2.8 | 732 | 1.16E-4 | 1.983 | AND GATE |
| pRHAB-(strong RBS) | 45 | 5906 | 4.31E-4 | 0.914 | OR GATE |
| pRHAB-(weak RBS) | 4.6 | 783 | 4.31E-4 | 0.914 | AND GATE |

Normalised outputs of a hypothetical AND gate design were modelled with transfer functions from 2 input switch devices of varying expression strength as listed in Supplementary Table 3. Supplementary Fig. 11 shows the predicted output of a hypothetical HrpRS AND gate. The model agreed well with experimental results (Fig. 2B) with a R^2^ value of 0.94.


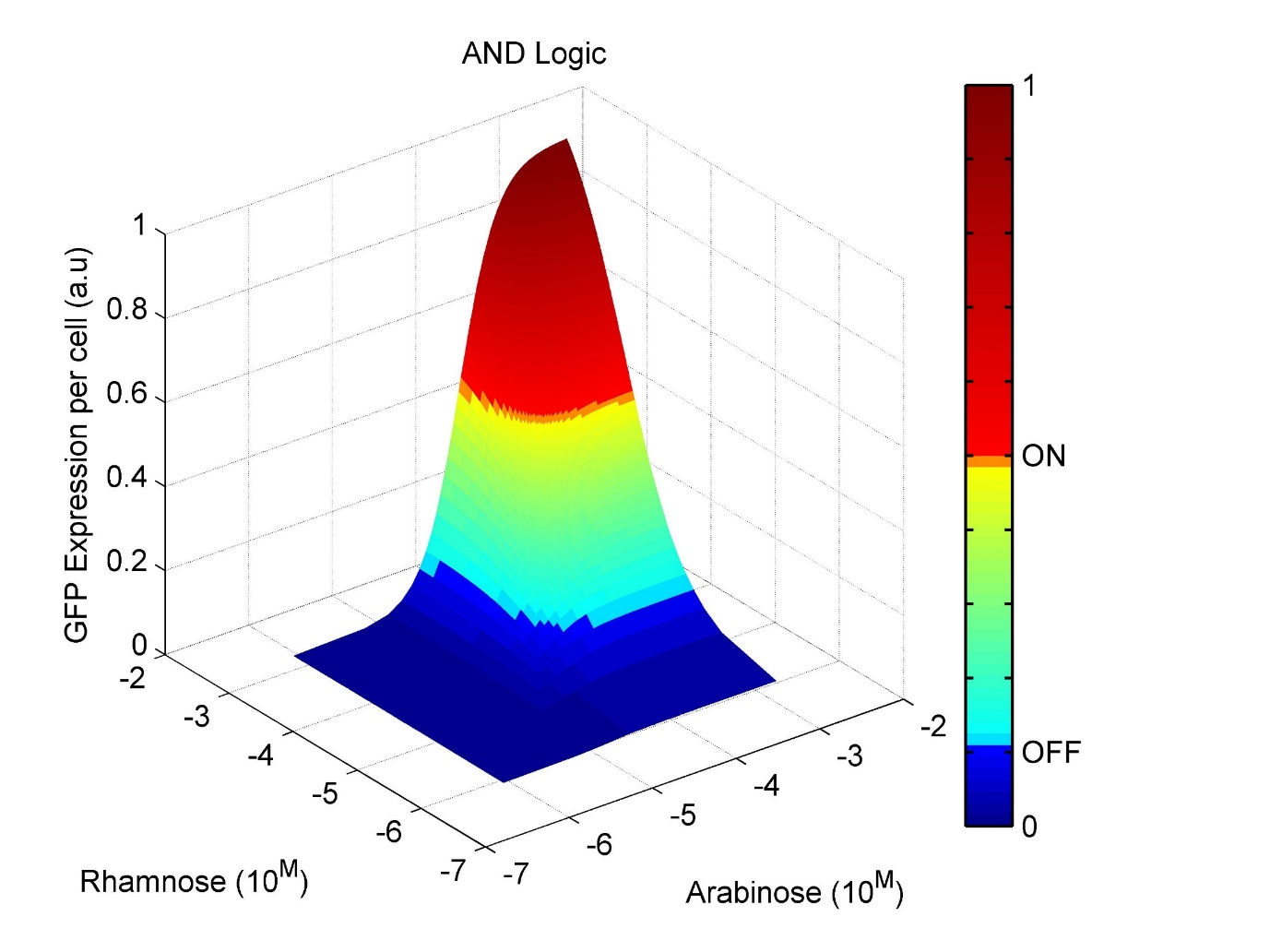


**Supplementary Fig. 11.** Predicted normalised output of HrpRS AND gate. The model was validated with a R^2^ value of 0.94.

To model an OR logic gate, transfer function of individual switch devices were incorporated to the following equation similar to how the effective resistance of serial arrangement of resistors was calculated.

$$\mathrm{OR}_{\mathrm{Abs}} = \left[ \alpha_{\mathrm{OR}} \left( TF1+TF2 \right) \right] \mathbf{Eqn.} (8)$$

Substituting, Eqn. (5) and (6) into Eqn. (8), the normalised output of OR logic gate:

$$\mathrm{OR}_{\mathrm{Norm}}= \frac{\left[ \alpha_{\mathrm{OR}} \left( TF1+TF2 \right) \right]}{\lim_{X_{1}X_{2}\to\infty} \left[ \alpha_{\mathrm{OR}} \left( TF1+TF2 \right) \right]}$$

$$= \frac{\left( TF1+TF2 \right)}{\lim_{X_{1}X_{2}\to\infty} \left[ A_{1}+ \frac{B_{1}\left( {X_{1}}^{n1} \right)}{{C_{1}}^{n1}+ {X_{1}}^{n1}}+ A_{2}+ \frac{B_{2}\left( {X_{2}}^{n2} \right)}{{C_{2}}^{n2}+ {X_{2}}^{n2}} \right]} \mathbf{Eqn.} (9)$$

Thus, the normalised output of OR logic gate is derived as:

$$\mathrm{OR}_{\mathrm{Norm}} =\left[ \frac{\left( TF1+TF2 \right)}{B_{1}+B_{2}} \right] \mathbf{Eqn.}(10)$$

Normalised outputs of a hypothetical OR gate were modelled with transfer functions from 2 input switch devices of strong expression strength as listed in Supplementary Table 3. Fig. 12 shows the predicted output of a hypothetical pBAD-pRHAB OR gate. The model agreed well with experimental results (Fig. 3B) with a R^2^ value of 0.96.

**
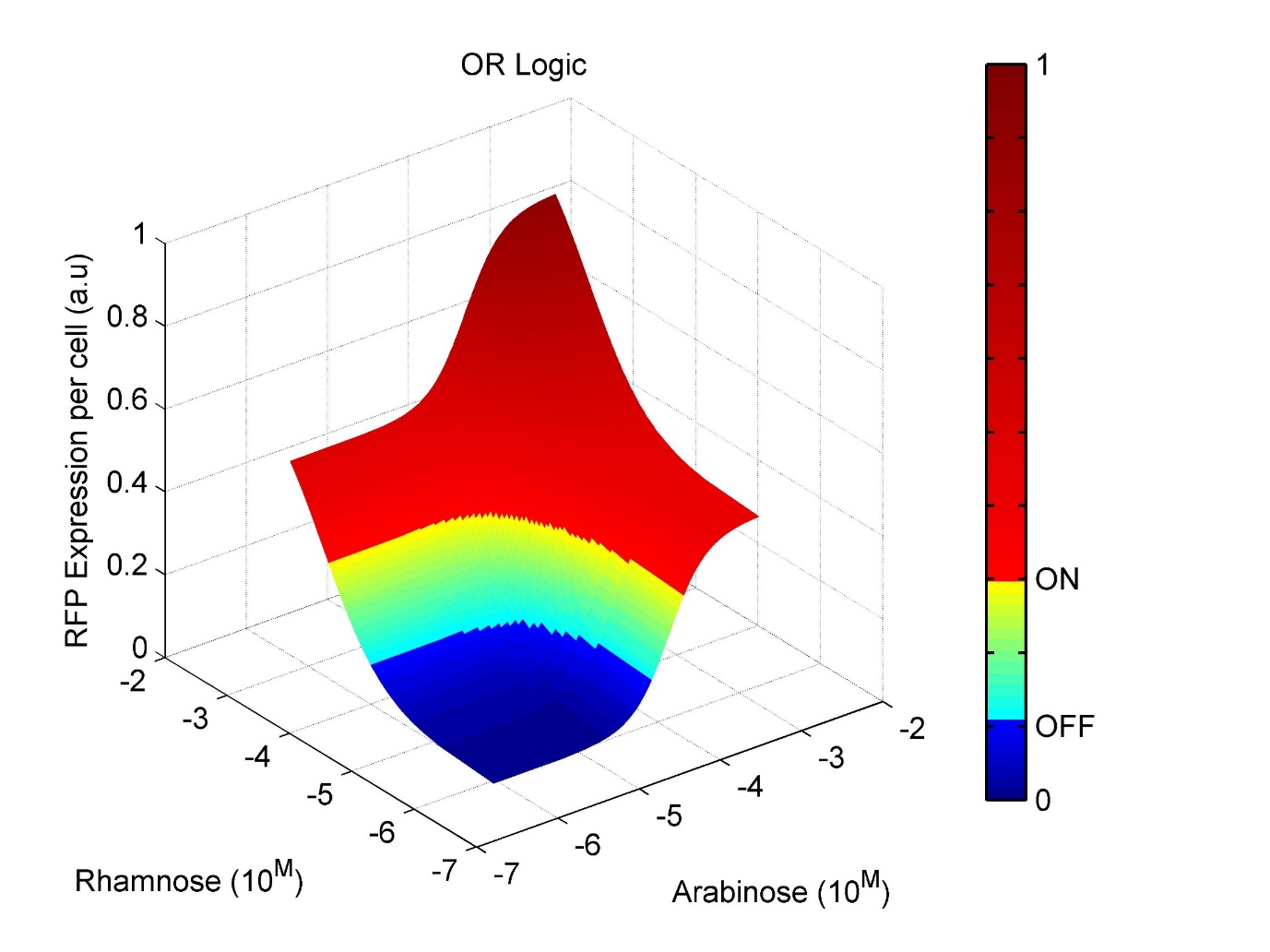
**

**Supplementary Fig. 12.** Predicted normalised output of pBAD-pRHAB OR gate. The model was validated with a R^2^ value of 0.96.


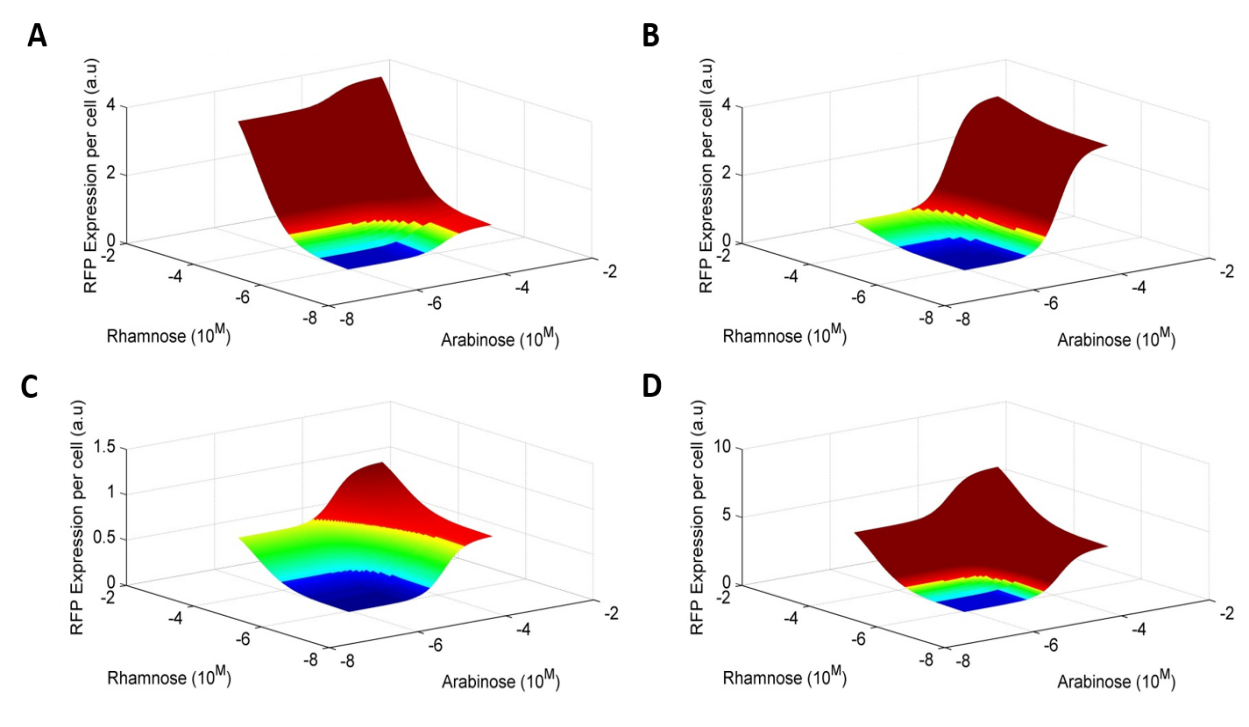


**Supplementary Fig. 13.** Predicted normalised outputs of 4 different OR gate designs with transfer functions corresponding to (A) TF1W-TF2S, (B) TF1S-TF2W, (C) TF1W-TF2W, and (D) TF1S-TF2S. From the model, only design C and D are functional OR gates, while design A and B are imbalanced in the overall expression profile.

The XOR logic gate was designed by using λCl repressors generated from HrpRS AND gate to inhibit output from the OR logic gate. Thus, outputs of XOR logic could be modelled as a difference of OR and AND outputs:

$$\mathrm{XOR}_{\mathrm{Abs}} = \mathrm{OR}_{\mathrm{Abs}}-\mathrm{AND}_{\mathrm{Abs}} \mathbf{Eqn.} (8)$$

In the presence of both input A and B, an ideal XOR logic would display an output of zero. Thus, in order for layered logic gates to perform XOR logic operation when both input A and B are introduced,

$$\mathrm{OR}_{\mathrm{Abs}}-\mathrm{AND}_{\mathrm{Abs}} \leq0$$

$$\mathrm{OR}_{\mathrm{Abs}}\leq\mathrm{AND}_{\mathrm{Abs}} (condition for XOR) \mathbf{Eqn.} (9)$$

The arabinose promoter had a maximum expression of ~20,000au GFP in the presence of input B (Supplementary Fig. 4B). Comparatively, pHrpL promoter in a high copy plasmid exhibited an expression of ~4000au GFP in the presence of both input A and B (Fig. 2D). By interpolating 4000au GFP expression on the Y-axis of the characterisation curve of pBAD promoter (Supplementary Fig. 4B), we estimated that the pHrpL promoter had an expression strength that corresponded to pBAD that was induced by 5.00E-05M arabinose on the X-axis. Accordingly, the NIMPLY gate (with strong RBS in OR gate component) in Fig. 4C shows that at 5.00E-05M arabinose and 28mM rhamnose, an RFP output of ~1775au was observed. That is, when pHrpL promoter was used to drive the production of λCl repressors, the percentage repression in the overall XOR logic was only ~37%, resulting in significant expression leakiness.

$$\% Repression=\frac{\mathrm{IMPLY}_{Abs, 0M arabinose}-\mathrm{IMPLY}_{Abs, 5.00E-05M arabinose}}{\mathrm{IMPLY}_{Abs, 0M arabinose}} \times100$$

$$=\frac{2830-1775}{2830} \times100=37\% \mathbf{Eqn.} (10)$$

From the above results, we reasoned that the OR gate that was originally constructed with strong RBS should be replaced with a weaker RBS so that the layered NOT gate will not be leaky. This fine-tuning procedure enabled the sequential layering of AND, OR and NOT logic gates to display robust XOR digital operation in single (bacterial) cell.

**Sequence Information**

blue: Promoter

Green: RBS

purple: Gene Coding Sequence

red: Terminator

Black: DNA SCAR

**Input Switch Device A with strong RbsA (pSB1C3, pMB1 replication origin)**

pCON.Rbs.RhaS.Rbs.AraC.T15-pBAD.RbsA.RFP.T15

tttacagctagctcagtcctaggtattatgctagcTACTAGAGGGAGCCCAAATGACCGTAttacatagtgtggatttttttccgtctggtaacgcgtccgtggcgatagaaccccggctcccgcaggcggattttcctgaacatcatcatgattttcatgaaattgtgattgtcgaacatggcacgggtattcatgtgtttaatgggcagccctataccatcaccggtggcacggtctgtttcgtacgcgatcatgatcggcatctgtatgaacataccgataatctgtgtctgaccaatgtgctgtatcgctcgccggatcgatttcagtttctcgccgggctgaatcagttgctgccacaagagctggatgggcagtatccgtctcactggcgcgttaaccacagcgtattgcagcaggtgcgacagctggttgcacagatggaacagcaggaaggggaaaatgatttaccctcgaccgccagtcgcgagatcttgtttatgcaattactgctcttgctgcgtaaaagcagtttgcaggagaacctggaaaacagcgcatcacgtctcaacttgcttctggcctggctggaggaccattttgccgatgaggtgaattgggatgccgtggcggatcaattttctctttcactgcgtacgctacatcggcagcttaagcagcaaacgggactgacgcctcagcgatacctgaaccgcctgcgactgatgaaagcccgacatctgctacgccacagcgaggccagcgttactgacatcgcctatcgctgtggattcagcgacagtaaccacttttcgacgctttttcgccgagagtttaactggtcaccgcgtgatattcgccagggacgggatggctttctgcaataaCGAGAGAGAGTTAACatggctgaagcgcaaaatgatcccctgctgccgggatactcgtttaacgcccatctggtggcgggtttaacgccgattgaggccaacggttatctcgatttttttatcgaccgaccgctgggaatgaaaggttatattctcaatctcaccattcgcggtcagggggtggtgaaaaatcagggacgagaatttgtctgccgaccgggtgatattttgctgttcccgccaggagagattcatcactacggtcgtcatccggaggctcgcgaatggtatcaccagtgggtttactttcgtccgcgcgcctactggcatgaatggcttaactggccgtcaatatttgccaatacgggtttctttcgcccggatgaagcgcaccagccgcatttcagcgacctgtttgggcaaatcattaacgccgggcaaggggaagggcgctattcggagctgctggcgataaatctgcttgagcaattgttactgcggcgcatggaagcgattaacgagtcgctccatccaccgatggataatcgggtacgcgaggcttgtcagtacatcagcgatcacctggcagacagcaattttgatatcgccagcgtcgcacagcatgtttgcttgtcgccgtcgcgtctgtcacatcttttccgccagcagttagggattagcgtcttaagctggcgcgaggaccaacgcattagtcaggcgaagctgcttttgagcactacccggatgcctatcgccaccgtcggtcgcaatgttggttttgacgatcaactctatttctcgcgagtatttaaaaaatgcaccggggccagcccgagcgagtttcgtgccggttgtgaagaaaaagtgaatgatgtagccgtcaagttgtcataaccaggcatcaaataaaacgaaaggctcagtcgaaagactgggcctttcgttttatctgttgtttgtcggtgaacgctctctactagagtcacactggctcaccttcgggtgggcctttctgcgtttataTACTAGAGagaagaaaccaattgtccatattgcatcagacattgccgtcactgcgtcttttactggctcttctcgctaaccaaaccggtaaccccgcttattaaaagcattctgtaacaaagcgggaccaaagccatgacaaaaacgcgtaacaaaagtgtctataatcacggcagaaaagtccacattgattatttgcacggcgtcacactttgctatgccatagcatttttatccataagattagcggattctacctgacgctttttatcgcaactctctactgtttctccaT**A**TACTAGAGAAAGAGGAGAAAATGGCTTCCTCCGAAGACGTTATCAAAGAGTTCATGCGTTTCAAAGTTCGTATGGAAGGTTCCGTTAACGGTCACGAGTTCGAAATCGAAGGTGAAGGTGAAGGTCGTCCGTACGAAGGTACCCAGACCGCTAAACTGAAAGTTACCAAAGGTGGTCCGCTGCCGTTCGCTTGGGACATCCTGTCCCCGCAGTTCCAGTACGGTTCCAAAGCTTACGTTAAACACCCGGCTGACATCCCGGACTACCTGAAACTGTCCTTCCCGGAAGGTTTCAAATGGGAACGTGTTATGAACTTCGAAGACGGTGGTGTTGTTACCGTTACCCAGGACTCCTCCCTGCAAGACGGTGAGTTCATCTACAAAGTTAAACTGCGTGGTACCAACTTCCCGTCCGACGGTCCGGTTATGCAGAAAAAAACCATGGGTTGGGAAGCTTCCACCGAACGTATGTACCCGGAAGACGGTGCTCTGAAAGGTGAAATCAAAATGCGTCTGAAACTGAAAGACGGTGGTCACTACGACGCTGAAGTTAAAACCACCTACATGGCTAAAAAACCGGTTCAGCTGCCGGGTGCTTACAAAACCGACATCAAACTGGACATCACCTCCCACAACGAAGACTACACCATCGTTGAACAGTACGAACGTGCTGAAGGTCGTCACTCCACCGGTGCTTAATAATACTAGAGCCAGGCATCAAATAAAACGAAAGGCTCAGTCGAAAGACTGGGCCTTTCGTTTTATCTGTTGTTTGTCGGTGAACGCTCTCTACTAGAGTCACACTGGCTCACCTTCGGGTGGGCCTTTCTGCGTTTATA

**Input Switch Device A with weak RbsA (pSB1C3, pMB1 replication origin)**

pCON.Rbs.RhaS.Rbs.AraC.T15-pBAD.RbsB.RFP.T15

tttacagctagctcagtcctaggtattatgctagcTACTAGAGGGAGCCCAAATGACCGTAttacatagtgtggatttttttccgtctggtaacgcgtccgtggcgatagaaccccggctcccgcaggcggattttcctgaacatcatcatgattttcatgaaattgtgattgtcgaacatggcacgggtattcatgtgtttaatgggcagccctataccatcaccggtggcacggtctgtttcgtacgcgatcatgatcggcatctgtatgaacataccgataatctgtgtctgaccaatgtgctgtatcgctcgccggatcgatttcagtttctcgccgggctgaatcagttgctgccacaagagctggatgggcagtatccgtctcactggcgcgttaaccacagcgtattgcagcaggtgcgacagctggttgcacagatggaacagcaggaaggggaaaatgatttaccctcgaccgccagtcgcgagatcttgtttatgcaattactgctcttgctgcgtaaaagcagtttgcaggagaacctggaaaacagcgcatcacgtctcaacttgcttctggcctggctggaggaccattttgccgatgaggtgaattgggatgccgtggcggatcaattttctctttcactgcgtacgctacatcggcagcttaagcagcaaacgggactgacgcctcagcgatacctgaaccgcctgcgactgatgaaagcccgacatctgctacgccacagcgaggccagcgttactgacatcgcctatcgctgtggattcagcgacagtaaccacttttcgacgctttttcgccgagagtttaactggtcaccgcgtgatattcgccagggacgggatggctttctgcaataaCGAGAGAGAGTTAACatggctgaagcgcaaaatgatcccctgctgccgggatactcgtttaacgcccatctggtggcgggtttaacgccgattgaggccaacggttatctcgatttttttatcgaccgaccgctgggaatgaaaggttatattctcaatctcaccattcgcggtcagggggtggtgaaaaatcagggacgagaatttgtctgccgaccgggtgatattttgctgttcccgccaggagagattcatcactacggtcgtcatccggaggctcgcgaatggtatcaccagtgggtttactttcgtccgcgcgcctactggcatgaatggcttaactggccgtcaatatttgccaatacgggtttctttcgcccggatgaagcgcaccagccgcatttcagcgacctgtttgggcaaatcattaacgccgggcaaggggaagggcgctattcggagctgctggcgataaatctgcttgagcaattgttactgcggcgcatggaagcgattaacgagtcgctccatccaccgatggataatcgggtacgcgaggcttgtcagtacatcagcgatcacctggcagacagcaattttgatatcgccagcgtcgcacagcatgtttgcttgtcgccgtcgcgtctgtcacatcttttccgccagcagttagggattagcgtcttaagctggcgcgaggaccaacgcattagtcaggcgaagctgcttttgagcactacccggatgcctatcgccaccgtcggtcgcaatgttggttttgacgatcaactctatttctcgcgagtatttaaaaaatgcaccggggccagcccgagcgagtttcgtgccggttgtgaagaaaaagtgaatgatgtagccgtcaagttgtcataaccaggcatcaaataaaacgaaaggctcagtcgaaagactgggcctttcgttttatctgttgtttgtcggtgaacgctctctactagagtcacactggctcaccttcgggtgggcctttctgcgtttataTACTAGAGagaagaaaccaattgtccatattgcatcagacattgccgtcactgcgtcttttactggctcttctcgctaaccaaaccggtaaccccgcttattaaaagcattctgtaacaaagcgggaccaaagccatgacaaaaacgcgtaacaaaagtgtctataatcacggcagaaaagtccacattgattatttgcacggcgtcacactttgctatgccatagcatttttatccataagattagcggattctacctgacgctttttatcgcaactctctactgtttctccaT**A**TACTAGAGTCACACAGGAAAGTACTAGATGGCTTCCTCCGAAGACGTTATCAAAGAGTTCATGCGTTTCAAAGTTCGTATGGAAGGTTCCGTTAACGGTCACGAGTTCGAAATCGAAGGTGAAGGTGAAGGTCGTCCGTACGAAGGTACCCAGACCGCTAAACTGAAAGTTACCAAAGGTGGTCCGCTGCCGTTCGCTTGGGACATCCTGTCCCCGCAGTTCCAGTACGGTTCCAAAGCTTACGTTAAACACCCGGCTGACATCCCGGACTACCTGAAACTGTCCTTCCCGGAAGGTTTCAAATGGGAACGTGTTATGAACTTCGAAGACGGTGGTGTTGTTACCGTTACCCAGGACTCCTCCCTGCAAGACGGTGAGTTCATCTACAAAGTTAAACTGCGTGGTACCAACTTCCCGTCCGACGGTCCGGTTATGCAGAAAAAAACCATGGGTTGGGAAGCTTCCACCGAACGTATGTACCCGGAAGACGGTGCTCTGAAAGGTGAAATCAAAATGCGTCTGAAACTGAAAGACGGTGGTCACTACGACGCTGAAGTTAAAACCACCTACATGGCTAAAAAACCGGTTCAGCTGCCGGGTGCTTACAAAACCGACATCAAACTGGACATCACCTCCCACAACGAAGACTACACCATCGTTGAACAGTACGAACGTGCTGAAGGTCGTCACTCCACCGGTGCTTAATAATACTAGAGCCAGGCATCAAATAAAACGAAAGGCTCAGTCGAAAGACTGGGCCTTTCGTTTTATCTGTTGTTTGTCGGTGAACGCTCTCTACTAGAGTCACACTGGCTCACCTTCGGGTGGGCCTTTCTGCGTTTATA

**Input Switch Device B with strong RbsA (pSB1A3, pMB1 replication origin)**

pCON.Rbs.RhaS.Rbs.AraC.T15-pRHAB.RbsA.RFP.T15

tttacagctagctcagtcctaggtattatgctagcTACTAGAGGGAGCCCAAATGACCGTAttacatagtgtggatttttttccgtctggtaacgcgtccgtggcgatagaaccccggctcccgcaggcggattttcctgaacatcatcatgattttcatgaaattgtgattgtcgaacatggcacgggtattcatgtgtttaatgggcagccctataccatcaccggtggcacggtctgtttcgtacgcgatcatgatcggcatctgtatgaacataccgataatctgtgtctgaccaatgtgctgtatcgctcgccggatcgatttcagtttctcgccgggctgaatcagttgctgccacaagagctggatgggcagtatccgtctcactggcgcgttaaccacagcgtattgcagcaggtgcgacagctggttgcacagatggaacagcaggaaggggaaaatgatttaccctcgaccgccagtcgcgagatcttgtttatgcaattactgctcttgctgcgtaaaagcagtttgcaggagaacctggaaaacagcgcatcacgtctcaacttgcttctggcctggctggaggaccattttgccgatgaggtgaattgggatgccgtggcggatcaattttctctttcactgcgtacgctacatcggcagcttaagcagcaaacgggactgacgcctcagcgatacctgaaccgcctgcgactgatgaaagcccgacatctgctacgccacagcgaggccagcgttactgacatcgcctatcgctgtggattcagcgacagtaaccacttttcgacgctttttcgccgagagtttaactggtcaccgcgtgatattcgccagggacgggatggctttctgcaataaCGAGAGAGAGTTAACatggctgaagcgcaaaatgatcccctgctgccgggatactcgtttaacgcccatctggtggcgggtttaacgccgattgaggccaacggttatctcgatttttttatcgaccgaccgctgggaatgaaaggttatattctcaatctcaccattcgcggtcagggggtggtgaaaaatcagggacgagaatttgtctgccgaccgggtgatattttgctgttcccgccaggagagattcatcactacggtcgtcatccggaggctcgcgaatggtatcaccagtgggtttactttcgtccgcgcgcctactggcatgaatggcttaactggccgtcaatatttgccaatacgggtttctttcgcccggatgaagcgcaccagccgcatttcagcgacctgtttgggcaaatcattaacgccgggcaaggggaagggcgctattcggagctgctggcgataaatctgcttgagcaattgttactgcggcgcatggaagcgattaacgagtcgctccatccaccgatggataatcgggtacgcgaggcttgtcagtacatcagcgatcacctggcagacagcaattttgatatcgccagcgtcgcacagcatgtttgcttgtcgccgtcgcgtctgtcacatcttttccgccagcagttagggattagcgtcttaagctggcgcgaggaccaacgcattagtcaggcgaagctgcttttgagcactacccggatgcctatcgccaccgtcggtcgcaatgttggttttgacgatcaactctatttctcgcgagtatttaaaaaatgcaccggggccagcccgagcgagtttcgtgccggttgtgaagaaaaagtgaatgatgtagccgtcaagttgtcataaccaggcatcaaataaaacgaaaggctcagtcgaaagactgggcctttcgttttatctgttgtttgtcggtgaacgctctctactagagtcacactggctcaccttcgggtgggcctttctgcgtttataTACTAGAGAGACCTTTACGCCGCTGGAGCAGGAATGCGGTGAGCATCACATCACCACAATTCAGCAAATTGTGAACATCATCACGTTCATCTTTCCCTGGTTGCCAATGGCCCATTTTCCTGTCAGTAACGAGAAGGTCGCGAATTGAGGCGCTTTTTAGACTGGTCGT**A**TACTAGAGAAAGAGGAGAAAATGGCTTCCTCCGAAGACGTTATCAAAGAGTTCATGCGTTTCAAAGTTCGTATGGAAGGTTCCGTTAACGGTCACGAGTTCGAAATCGAAGGTGAAGGTGAAGGTCGTCCGTACGAAGGTACCCAGACCGCTAAACTGAAAGTTACCAAAGGTGGTCCGCTGCCGTTCGCTTGGGACATCCTGTCCCCGCAGTTCCAGTACGGTTCCAAAGCTTACGTTAAACACCCGGCTGACATCCCGGACTACCTGAAACTGTCCTTCCCGGAAGGTTTCAAATGGGAACGTGTTATGAACTTCGAAGACGGTGGTGTTGTTACCGTTACCCAGGACTCCTCCCTGCAAGACGGTGAGTTCATCTACAAAGTTAAACTGCGTGGTACCAACTTCCCGTCCGACGGTCCGGTTATGCAGAAAAAAACCATGGGTTGGGAAGCTTCCACCGAACGTATGTACCCGGAAGACGGTGCTCTGAAAGGTGAAATCAAAATGCGTCTGAAACTGAAAGACGGTGGTCACTACGACGCTGAAGTTAAAACCACCTACATGGCTAAAAAACCGGTTCAGCTGCCGGGTGCTTACAAAACCGACATCAAACTGGACATCACCTCCCACAACGAAGACTACACCATCGTTGAACAGTACGAACGTGCTGAAGGTCGTCACTCCACCGGTGCTTAATAATACTAGAGCCAGGCATCAAATAAAACGAAAGGCTCAGTCGAAAGACTGGGCCTTTCGTTTTATCTGTTGTTTGTCGGTGAACGCTCTCTACTAGAGTCACACTGGCTCACCTTCGGGTGGGCCTTTCTGCGTTTATA

**Input Switch Device B with weak RbsB (pSB1A3, pMB1 replication origin)**

pCON.Rbs.RhaS.Rbs.AraC.T15-pRHAB.RbsB.RFP.T15

tttacagctagctcagtcctaggtattatgctagcTACTAGAGGGAGCCCAAATGACCGTAttacatagtgtggatttttttccgtctggtaacgcgtccgtggcgatagaaccccggctcccgcaggcggattttcctgaacatcatcatgattttcatgaaattgtgattgtcgaacatggcacgggtattcatgtgtttaatgggcagccctataccatcaccggtggcacggtctgtttcgtacgcgatcatgatcggcatctgtatgaacataccgataatctgtgtctgaccaatgtgctgtatcgctcgccggatcgatttcagtttctcgccgggctgaatcagttgctgccacaagagctggatgggcagtatccgtctcactggcgcgttaaccacagcgtattgcagcaggtgcgacagctggttgcacagatggaacagcaggaaggggaaaatgatttaccctcgaccgccagtcgcgagatcttgtttatgcaattactgctcttgctgcgtaaaagcagtttgcaggagaacctggaaaacagcgcatcacgtctcaacttgcttctggcctggctggaggaccattttgccgatgaggtgaattgggatgccgtggcggatcaattttctctttcactgcgtacgctacatcggcagcttaagcagcaaacgggactgacgcctcagcgatacctgaaccgcctgcgactgatgaaagcccgacatctgctacgccacagcgaggccagcgttactgacatcgcctatcgctgtggattcagcgacagtaaccacttttcgacgctttttcgccgagagtttaactggtcaccgcgtgatattcgccagggacgggatggctttctgcaataaCGAGAGAGAGTTAACatggctgaagcgcaaaatgatcccctgctgccgggatactcgtttaacgcccatctggtggcgggtttaacgccgattgaggccaacggttatctcgatttttttatcgaccgaccgctgggaatgaaaggttatattctcaatctcaccattcgcggtcagggggtggtgaaaaatcagggacgagaatttgtctgccgaccgggtgatattttgctgttcccgccaggagagattcatcactacggtcgtcatccggaggctcgcgaatggtatcaccagtgggtttactttcgtccgcgcgcctactggcatgaatggcttaactggccgtcaatatttgccaatacgggtttctttcgcccggatgaagcgcaccagccgcatttcagcgacctgtttgggcaaatcattaacgccgggcaaggggaagggcgctattcggagctgctggcgataaatctgcttgagcaattgttactgcggcgcatggaagcgattaacgagtcgctccatccaccgatggataatcgggtacgcgaggcttgtcagtacatcagcgatcacctggcagacagcaattttgatatcgccagcgtcgcacagcatgtttgcttgtcgccgtcgcgtctgtcacatcttttccgccagcagttagggattagcgtcttaagctggcgcgaggaccaacgcattagtcaggcgaagctgcttttgagcactacccggatgcctatcgccaccgtcggtcgcaatgttggttttgacgatcaactctatttctcgcgagtatttaaaaaatgcaccggggccagcccgagcgagtttcgtgccggttgtgaagaaaaagtgaatgatgtagccgtcaagttgtcataaccaggcatcaaataaaacgaaaggctcagtcgaaagactgggcctttcgttttatctgttgtttgtcggtgaacgctctctactagagtcacactggctcaccttcgggtgggcctttctgcgtttataTACTAGAGAGACCTTTACGCCGCTGGAGCAGGAATGCGGTGAGCATCACATCACCACAATTCAGCAAATTGTGAACATCATCACGTTCATCTTTCCCTGGTTGCCAATGGCCCATTTTCCTGTCAGTAACGAGAAGGTCGCGAATTGAGGCGCTTTTTAGACTGGTCGT**A**TACTAGAGTCACACAGGAAAGTACTAGATGGCTTCCTCCGAAGACGTTATCAAAGAGTTCATGCGTTTCAAAGTTCGTATGGAAGGTTCCGTTAACGGTCACGAGTTCGAAATCGAAGGTGAAGGTGAAGGTCGTCCGTACGAAGGTACCCAGACCGCTAAACTGAAAGTTACCAAAGGTGGTCCGCTGCCGTTCGCTTGGGACATCCTGTCCCCGCAGTTCCAGTACGGTTCCAAAGCTTACGTTAAACACCCGGCTGACATCCCGGACTACCTGAAACTGTCCTTCCCGGAAGGTTTCAAATGGGAACGTGTTATGAACTTCGAAGACGGTGGTGTTGTTACCGTTACCCAGGACTCCTCCCTGCAAGACGGTGAGTTCATCTACAAAGTTAAACTGCGTGGTACCAACTTCCCGTCCGACGGTCCGGTTATGCAGAAAAAAACCATGGGTTGGGAAGCTTCCACCGAACGTATGTACCCGGAAGACGGTGCTCTGAAAGGTGAAATCAAAATGCGTCTGAAACTGAAAGACGGTGGTCACTACGACGCTGAAGTTAAAACCACCTACATGGCTAAAAAACCGGTTCAGCTGCCGGGTGCTTACAAAACCGACATCAAACTGGACATCACCTCCCACAACGAAGACTACACCATCGTTGAACAGTACGAACGTGCTGAAGGTCGTCACTCCACCGGTGCTTAATAATACTAGAGCCAGGCATCAAATAAAACGAAAGGCTCAGTCGAAAGACTGGGCCTTTCGTTTTATCTGTTGTTTGTCGGTGAACGCTCTCTACTAGAGTCACACTGGCTCACCTTCGGGTGGGCCTTTCTGCGTTTATA

**Pairwise Compatibility Test Construct (pSB1A3, pMB1 replication origin)**

pCON.Rbs.RhaS.Rbs.AraC.T15-pRHAB.RbsA.RFP.T15-pBAD.RbsA2.GFP.T15

tttacagctagctcagtcctaggtattatgctagcTACTAGAGGGAGCCCAAATGACCGTAttacatagtgtggatttttttccgtctggtaacgcgtccgtggcgatagaaccccggctcccgcaggcggattttcctgaacatcatcatgattttcatgaaattgtgattgtcgaacatggcacgggtattcatgtgtttaatgggcagccctataccatcaccggtggcacggtctgtttcgtacgcgatcatgatcggcatctgtatgaacataccgataatctgtgtctgaccaatgtgctgtatcgctcgccggatcgatttcagtttctcgccgggctgaatcagttgctgccacaagagctggatgggcagtatccgtctcactggcgcgttaaccacagcgtattgcagcaggtgcgacagctggttgcacagatggaacagcaggaaggggaaaatgatttaccctcgaccgccagtcgcgagatcttgtttatgcaattactgctcttgctgcgtaaaagcagtttgcaggagaacctggaaaacagcgcatcacgtctcaacttgcttctggcctggctggaggaccattttgccgatgaggtgaattgggatgccgtggcggatcaattttctctttcactgcgtacgctacatcggcagcttaagcagcaaacgggactgacgcctcagcgatacctgaaccgcctgcgactgatgaaagcccgacatctgctacgccacagcgaggccagcgttactgacatcgcctatcgctgtggattcagcgacagtaaccacttttcgacgctttttcgccgagagtttaactggtcaccgcgtgatattcgccagggacgggatggctttctgcaataaCGAGAGAGAGTTAACatggctgaagcgcaaaatgatcccctgctgccgggatactcgtttaacgcccatctggtggcgggtttaacgccgattgaggccaacggttatctcgatttttttatcgaccgaccgctgggaatgaaaggttatattctcaatctcaccattcgcggtcagggggtggtgaaaaatcagggacgagaatttgtctgccgaccgggtgatattttgctgttcccgccaggagagattcatcactacggtcgtcatccggaggctcgcgaatggtatcaccagtgggtttactttcgtccgcgcgcctactggcatgaatggcttaactggccgtcaatatttgccaatacgggtttctttcgcccggatgaagcgcaccagccgcatttcagcgacctgtttgggcaaatcattaacgccgggcaaggggaagggcgctattcggagctgctggcgataaatctgcttgagcaattgttactgcggcgcatggaagcgattaacgagtcgctccatccaccgatggataatcgggtacgcgaggcttgtcagtacatcagcgatcacctggcagacagcaattttgatatcgccagcgtcgcacagcatgtttgcttgtcgccgtcgcgtctgtcacatcttttccgccagcagttagggattagcgtcttaagctggcgcgaggaccaacgcattagtcaggcgaagctgcttttgagcactacccggatgcctatcgccaccgtcggtcgcaatgttggttttgacgatcaactctatttctcgcgagtatttaaaaaatgcaccggggccagcccgagcgagtttcgtgccggttgtgaagaaaaagtgaatgatgtagccgtcaagttgtcataaccaggcatcaaataaaacgaaaggctcagtcgaaagactgggcctttcgttttatctgttgtttgtcggtgaacgctctctactagagtcacactggctcaccttcgggtgggcctttctgcgtttataTACTAGAGAGACCTTTACGCCGCTGGAGCAGGAATGCGGTGAGCATCACATCACCACAATTCAGCAAATTGTGAACATCATCACGTTCATCTTTCCCTGGTTGCCAATGGCCCATTTTCCTGTCAGTAACGAGAAGGTCGCGAATTGAGGCGCTTTTTAGACTGGTCGT**A**TACTAGAGAAAGAGGAGAAAATGGCTTCCTCCGAAGACGTTATCAAAGAGTTCATGCGTTTCAAAGTTCGTATGGAAGGTTCCGTTAACGGTCACGAGTTCGAAATCGAAGGTGAAGGTGAAGGTCGTCCGTACGAAGGTACCCAGACCGCTAAACTGAAAGTTACCAAAGGTGGTCCGCTGCCGTTCGCTTGGGACATCCTGTCCCCGCAGTTCCAGTACGGTTCCAAAGCTTACGTTAAACACCCGGCTGACATCCCGGACTACCTGAAACTGTCCTTCCCGGAAGGTTTCAAATGGGAACGTGTTATGAACTTCGAAGACGGTGGTGTTGTTACCGTTACCCAGGACTCCTCCCTGCAAGACGGTGAGTTCATCTACAAAGTTAAACTGCGTGGTACCAACTTCCCGTCCGACGGTCCGGTTATGCAGAAAAAAACCATGGGTTGGGAAGCTTCCACCGAACGTATGTACCCGGAAGACGGTGCTCTGAAAGGTGAAATCAAAATGCGTCTGAAACTGAAAGACGGTGGTCACTACGACGCTGAAGTTAAAACCACCTACATGGCTAAAAAACCGGTTCAGCTGCCGGGTGCTTACAAAACCGACATCAAACTGGACATCACCTCCCACAACGAAGACTACACCATCGTTGAACAGTACGAACGTGCTGAAGGTCGTCACTCCACCGGTGCTTAATAATACTAGAGCCAGGCATCAAATAAAACGAAAGGCTCAGTCGAAAGACTGGGCCTTTCGTTTTATCTGTTGTTTGTCGGTGAACGCTCTCTACTAGAGTCACACTGGCTCACCTTCGGGTGGGCCTTTCTGCGTTTATATACTAGAGagaagaaaccaattgtccatattgcatcagacattgccgtcactgcgtcttttactggctcttctcgctaaccaaaccggtaaccccgcttattaaaagcattctgtaacaaagcgggaccaaagccatgacaaaaacgcgtaacaaaagtgtctataatcacggcagaaaagtccacattgattatttgcacggcgtcacactttgctatgccatagcatttttatccataagattagcggattctacctgacgctttttatcgcaactctctactgtttctccaT**A**TACTAGAGGGAGCCCAATACTAGATGCGTAAAGGAGAAGAACTTTTCACTGGAGTTGTCCCAATTCTTGTTGAATTAGATGGTGATGTTAATGGGCACAAATTTTCTGTCAGTGGAGAGGGTGAAGGTGATGCAACATACGGAAAACTTACCCTTAAATTTATTTGCACTACTGGAAAACTACCTGTTCCATGGCCAACACTTGTCACTACTTTCGGTTATGGTGTTCAATGCTTTGCGAGATACCCAGATCATATGAAACAGCATGACTTTTTCAAGAGTGCCATGCCCGAAGGTTATGTACAGGAAAGAACTATATTTTTCAAAGATGACGGGAACTACAAGACACGTGCTGAAGTCAAGTTTGAAGGTGATACCCTTGTTAATAGAATCGAGTTAAAAGGTATTGATTTTAAAGAAGATGGAAACATTCTTGGACACAAATTGGAATACAACTATAACTCACACAATGTATACATCATGGCAGACAAACAAAAGAATGGAATCAAAGTTAACTTCAAAATTAGACACAACATTGAAGATGGAAGCGTTCAACTAGCAGACCATTATCAACAAAATACTCCAATTGGCGATGGCCCTGTCCTTTTACCAGACAACCATTACCTGTCCACACAATCTGCCCTTTCGAAAGATCCCAACGAAAAGAGAGACCACATGGTCCTTCTTGAGTTTGTAACAGCTGCTGGGATTACACATGGCATGGATGAACTATACAAATAATAA

**σ54 Promoter Genetic Context Effect Test (pSB1C3, pMB1 origin or pSB4A5, pSC101 origin)**

pHrpL.Rbs.GFP.T15

GCCGGATTATGTCCGCTGAGTGGGTCACGGTCCCGGATCAGTTCCCTTGCGAAGCTGACCGATGTTTTTGTGCCAAAAGCTGTTGTGGCAAAAAACGGTTTGCGCAAAGTTTTGTATTACAAAGAATTTCACATTTTAAAATATCTTTATAAATCAATCAGTTATTTCTATTTTTAAGCTGGCATGGTTATCGCTATAGGGCTTGTACTACTAGAGATTAAAGAGGAGAAATACTAGATGCGTAAAGGAGAAGAACTTTTCACTGGAGTTGTCCCAATTCTTGTTGAATTAGATGGTGATGTTAATGGGCACAAATTTTCTGTCAGTGGAGAGGGTGAAGGTGATGCAACATACGGAAAACTTACCCTTAAATTTATTTGCACTACTGGAAAACTACCTGTTCCATGGCCAACACTTGTCACTACTTTCGGTTATGGTGTTCAATGCTTTGCGAGATACCCAGATCATATGAAACAGCATGACTTTTTCAAGAGTGCCATGCCCGAAGGTTATGTACAGGAAAGAACTATATTTTTCAAAGATGACGGGAACTACAAGACACGTGCTGAAGTCAAGTTTGAAGGTGATACCCTTGTTAATAGAATCGAGTTAAAAGGTATTGATTTTAAAGAAGATGGAAACATTCTTGGACACAAATTGGAATACAACTATAACTCACACAATGTATACATCATGGCAGACAAACAAAAGAATGGAATCAAAGTTAACTTCAAAATTAGACACAACATTGAAGATGGAAGCGTTCAACTAGCAGACCATTATCAACAAAATACTCCAATTGGCGATGGCCCTGTCCTTTTACCAGACAACCATTACCTGTCCACACAATCTGCCCTTTCGAAAGATCCCAACGAAAAGAGAGACCACATGGTCCTTCTTGAGTTTGTAACAGCTGCTGGGATTACACATGGCATGGATGAACTATACAAATAATAATACTAGAGCCAGGCATCAAATAAAACGAAAGGCTCAGTCGAAAGACTGGGCCTTTCGTTTTATCTGTTGTTTGTCGGTGAACGCTCTCTACTAGAGTCACACTGGCTCACCTTCGGGTGGGCCTTTCTGCGTTTATA

pHrpL.Rbs.GFP.T15-pHrpL.Rbs.λCl.T15

GCCGGATTATGTCCGCTGAGTGGGTCACGGTCCCGGATCAGTTCCCTTGCGAAGCTGACCGATGTTTTTGTGCCAAAAGCTGTTGTGGCAAAAAACGGTTTGCGCAAAGTTTTGTATTACAAAGAATTTCACATTTTAAAATATCTTTATAAATCAATCAGTTATTTCTATTTTTAAGCTGGCATGGTTATCGCTATAGGGCTTGTACTACTAGAGATTAAAGAGGAGAAATACTAGATGCGTAAAGGAGAAGAACTTTTCACTGGAGTTGTCCCAATTCTTGTTGAATTAGATGGTGATGTTAATGGGCACAAATTTTCTGTCAGTGGAGAGGGTGAAGGTGATGCAACATACGGAAAACTTACCCTTAAATTTATTTGCACTACTGGAAAACTACCTGTTCCATGGCCAACACTTGTCACTACTTTCGGTTATGGTGTTCAATGCTTTGCGAGATACCCAGATCATATGAAACAGCATGACTTTTTCAAGAGTGCCATGCCCGAAGGTTATGTACAGGAAAGAACTATATTTTTCAAAGATGACGGGAACTACAAGACACGTGCTGAAGTCAAGTTTGAAGGTGATACCCTTGTTAATAGAATCGAGTTAAAAGGTATTGATTTTAAAGAAGATGGAAACATTCTTGGACACAAATTGGAATACAACTATAACTCACACAATGTATACATCATGGCAGACAAACAAAAGAATGGAATCAAAGTTAACTTCAAAATTAGACACAACATTGAAGATGGAAGCGTTCAACTAGCAGACCATTATCAACAAAATACTCCAATTGGCGATGGCCCTGTCCTTTTACCAGACAACCATTACCTGTCCACACAATCTGCCCTTTCGAAAGATCCCAACGAAAAGAGAGACCACATGGTCCTTCTTGAGTTTGTAACAGCTGCTGGGATTACACATGGCATGGATGAACTATACAAATAATAATACTAGAGCCAGGCATCAAATAAAACGAAAGGCTCAGTCGAAAGACTGGGCCTTTCGTTTTATCTGTTGTTTGTCGGTGAACGCTCTCTACTAGAGTCACACTGGCTCACCTTCGGGTGGGCCTTTCTGCGTTTATATACTAGAGGCCGGATTATGTCCGCTGAGTGGGTCACGGTCCCGGATCAGTTCCCTTGCGAAGCTGACCGATGTTTTTGTGCCAAAAGCTGTTGTGGCAAAAAACGGTTTGCGCAAAGTTTTGTATTACAAAGAATTTCACATTTTAAAATATCTTTATAAATCAATCAGTTATTTCTATTTTTAAGCTGGCATGGTTATCGCTATAGGGCTTGTACTACTAGAAAAGAGGAGAAATACTAGATGAGCACAAAAAAGAAACCATTAACACAAGAGCAGCTTGAGGACGCACGTCGCCTTAAAGCAATTTATGAAAAAAAGAAAAATGAACTTGGCTTATCCCAGGAATCTGTCGCAGACAAGATGGGGATGGGGCAGTCAGGCGTTGGTGCTTTATTTAATGGCATCAATGCATTAAATGCTTATAACGCCGCATTGCTTGCAAAAATTCTCAAAGTTAGCGTTGAAGAATTTAGCCCTTCAATCGCCAGAGAAATCTACGAGATGTATGAAGCGGTTAGTATGCAGCCGTCACTTAGAAGTGAGTATGAGTACCCTGTTTTTTCTCATGTTCAGGCAGGGATGTTCTCACCTGAGCTTAGAACCTTTACCAAAGGTGATGCGGAGAGATGGGTAAGCACAACCAAAAAAGCCAGTGATTCTGCATTCTGGCTTGAGGTTGAAGGTAATTCCATGACCGCACCAACAGGCTCCAAGCCAAGCTTTCCTGACGGAATGTTAATTCTCGTTGACCCTGAGCAGGCTGTTGAGCCAGGTGATTTCTGCATAGCCAGACTTGGGGGTGATGAGTTTACCTTCAAGAAACTGATCAGGGATAGCGGTCAGGTGTTTTTACAACCACTAAACCCACAGTACCCAATGATCCCATGCAATGAGAGTTGTTCCGTTGTGGGGAAAGTTATCGCTAGTCAGTGGCCTGAAGAGACGTTTGGCTAATACTAGAGCCAGGCATCAAATAAAACGAAAGGCTCAGTCGAAAGACTGGGCCTTTCGTTTTATCTGTTGTTTGTCGGTGAACGCTCTCTACTAGAGTCACACTGGCTCACCTTCGGGTGGGCCTTTCTGCGTTTATA

pHrpL.Rbs.λCl.T15-pHrpL.Rbs.GFP.T15

GCCGGATTATGTCCGCTGAGTGGGTCACGGTCCCGGATCAGTTCCCTTGCGAAGCTGACCGATGTTTTTGTGCCAAAAGCTGTTGTGGCAAAAAACGGTTTGCGCAAAGTTTTGTATTACAAAGAATTTCACATTTTAAAATATCTTTATAAATCAATCAGTTATTTCTATTTTTAAGCTGGCATGGTTATCGCTATAGGGCTTGTACTACTAGAAAAGAGGAGAAATACTAGATGAGCACAAAAAAGAAACCATTAACACAAGAGCAGCTTGAGGACGCACGTCGCCTTAAAGCAATTTATGAAAAAAAGAAAAATGAACTTGGCTTATCCCAGGAATCTGTCGCAGACAAGATGGGGATGGGGCAGTCAGGCGTTGGTGCTTTATTTAATGGCATCAATGCATTAAATGCTTATAACGCCGCATTGCTTGCAAAAATTCTCAAAGTTAGCGTTGAAGAATTTAGCCCTTCAATCGCCAGAGAAATCTACGAGATGTATGAAGCGGTTAGTATGCAGCCGTCACTTAGAAGTGAGTATGAGTACCCTGTTTTTTCTCATGTTCAGGCAGGGATGTTCTCACCTGAGCTTAGAACCTTTACCAAAGGTGATGCGGAGAGATGGGTAAGCACAACCAAAAAAGCCAGTGATTCTGCATTCTGGCTTGAGGTTGAAGGTAATTCCATGACCGCACCAACAGGCTCCAAGCCAAGCTTTCCTGACGGAATGTTAATTCTCGTTGACCCTGAGCAGGCTGTTGAGCCAGGTGATTTCTGCATAGCCAGACTTGGGGGTGATGAGTTTACCTTCAAGAAACTGATCAGGGATAGCGGTCAGGTGTTTTTACAACCACTAAACCCACAGTACCCAATGATCCCATGCAATGAGAGTTGTTCCGTTGTGGGGAAAGTTATCGCTAGTCAGTGGCCTGAAGAGACGTTTGGCTAATACTAGAGCCAGGCATCAAATAAAACGAAAGGCTCAGTCGAAAGACTGGGCCTTTCGTTTTATCTGTTGTTTGTCGGTGAACGCTCTCTACTAGAGTCACACTGGCTCACCTTCGGGTGGGCCTTTCTGCGTTTATATACTAGAGGCCGGATTATGTCCGCTGAGTGGGTCACGGTCCCGGATCAGTTCCCTTGCGAAGCTGACCGATGTTTTTGTGCCAAAAGCTGTTGTGGCAAAAAACGGTTTGCGCAAAGTTTTGTATTACAAAGAATTTCACATTTTAAAATATCTTTATAAATCAATCAGTTATTTCTATTTTTAAGCTGGCATGGTTATCGCTATAGGGCTTGTACTACTAGAGATTAAAGAGGAGAAATACTAGATGCGTAAAGGAGAAGAACTTTTCACTGGAGTTGTCCCAATTCTTGTTGAATTAGATGGTGATGTTAATGGGCACAAATTTTCTGTCAGTGGAGAGGGTGAAGGTGATGCAACATACGGAAAACTTACCCTTAAATTTATTTGCACTACTGGAAAACTACCTGTTCCATGGCCAACACTTGTCACTACTTTCGGTTATGGTGTTCAATGCTTTGCGAGATACCCAGATCATATGAAACAGCATGACTTTTTCAAGAGTGCCATGCCCGAAGGTTATGTACAGGAAAGAACTATATTTTTCAAAGATGACGGGAACTACAAGACACGTGCTGAAGTCAAGTTTGAAGGTGATACCCTTGTTAATAGAATCGAGTTAAAAGGTATTGATTTTAAAGAAGATGGAAACATTCTTGGACACAAATTGGAATACAACTATAACTCACACAATGTATACATCATGGCAGACAAACAAAAGAATGGAATCAAAGTTAACTTCAAAATTAGACACAACATTGAAGATGGAAGCGTTCAACTAGCAGACCATTATCAACAAAATACTCCAATTGGCGATGGCCCTGTCCTTTTACCAGACAACCATTACCTGTCCACACAATCTGCCCTTTCGAAAGATCCCAACGAAAAGAGAGACCACATGGTCCTTCTTGAGTTTGTAACAGCTGCTGGGATTACACATGGCATGGATGAACTATACAAATAATAATACTAGAGCCAGGCATCAAATAAAACGAAAGGCTCAGTCGAAAGACTGGGCCTTTCGTTTTATCTGTTGTTTGTCGGTGAACGCTCTCTACTAGAGTCACACTGGCTCACCTTCGGGTGGGCCTTTCTGCGTTTATA

Rbs.λCl.T15-pHrpL.Rbs.GFP.T15

AAAGAGGAGAAATACTAGATGAGCACAAAAAAGAAACCATTAACACAAGAGCAGCTTGAGGACGCACGTCGCCTTAAAGCAATTTATGAAAAAAAGAAAAATGAACTTGGCTTATCCCAGGAATCTGTCGCAGACAAGATGGGGATGGGGCAGTCAGGCGTTGGTGCTTTATTTAATGGCATCAATGCATTAAATGCTTATAACGCCGCATTGCTTGCAAAAATTCTCAAAGTTAGCGTTGAAGAATTTAGCCCTTCAATCGCCAGAGAAATCTACGAGATGTATGAAGCGGTTAGTATGCAGCCGTCACTTAGAAGTGAGTATGAGTACCCTGTTTTTTCTCATGTTCAGGCAGGGATGTTCTCACCTGAGCTTAGAACCTTTACCAAAGGTGATGCGGAGAGATGGGTAAGCACAACCAAAAAAGCCAGTGATTCTGCATTCTGGCTTGAGGTTGAAGGTAATTCCATGACCGCACCAACAGGCTCCAAGCCAAGCTTTCCTGACGGAATGTTAATTCTCGTTGACCCTGAGCAGGCTGTTGAGCCAGGTGATTTCTGCATAGCCAGACTTGGGGGTGATGAGTTTACCTTCAAGAAACTGATCAGGGATAGCGGTCAGGTGTTTTTACAACCACTAAACCCACAGTACCCAATGATCCCATGCAATGAGAGTTGTTCCGTTGTGGGGAAAGTTATCGCTAGTCAGTGGCCTGAAGAGACGTTTGGCTAATACTAGAGCCAGGCATCAAATAAAACGAAAGGCTCAGTCGAAAGACTGGGCCTTTCGTTTTATCTGTTGTTTGTCGGTGAACGCTCTCTACTAGAGTCACACTGGCTCACCTTCGGGTGGGCCTTTCTGCGTTTATATACTAGAGGCCGGATTATGTCCGCTGAGTGGGTCACGGTCCCGGATCAGTTCCCTTGCGAAGCTGACCGATGTTTTTGTGCCAAAAGCTGTTGTGGCAAAAAACGGTTTGCGCAAAGTTTTGTATTACAAAGAATTTCACATTTTAAAATATCTTTATAAATCAATCAGTTATTTCTATTTTTAAGCTGGCATGGTTATCGCTATAGGGCTTGTACTACTAGAGATTAAAGAGGAGAAATACTAGATGCGTAAAGGAGAAGAACTTTTCACTGGAGTTGTCCCAATTCTTGTTGAATTAGATGGTGATGTTAATGGGCACAAATTTTCTGTCAGTGGAGAGGGTGAAGGTGATGCAACATACGGAAAACTTACCCTTAAATTTATTTGCACTACTGGAAAACTACCTGTTCCATGGCCAACACTTGTCACTACTTTCGGTTATGGTGTTCAATGCTTTGCGAGATACCCAGATCATATGAAACAGCATGACTTTTTCAAGAGTGCCATGCCCGAAGGTTATGTACAGGAAAGAACTATATTTTTCAAAGATGACGGGAACTACAAGACACGTGCTGAAGTCAAGTTTGAAGGTGATACCCTTGTTAATAGAATCGAGTTAAAAGGTATTGATTTTAAAGAAGATGGAAACATTCTTGGACACAAATTGGAATACAACTATAACTCACACAATGTATACATCATGGCAGACAAACAAAAGAATGGAATCAAAGTTAACTTCAAAATTAGACACAACATTGAAGATGGAAGCGTTCAACTAGCAGACCATTATCAACAAAATACTCCAATTGGCGATGGCCCTGTCCTTTTACCAGACAACCATTACCTGTCCACACAATCTGCCCTTTCGAAAGATCCCAACGAAAAGAGAGACCACATGGTCCTTCTTGAGTTTGTAACAGCTGCTGGGATTACACATGGCATGGATGAACTATACAAATAATAATACTAGAGCCAGGCATCAAATAAAACGAAAGGCTCAGTCGAAAGACTGGGCCTTTCGTTTTATCTGTTGTTTGTCGGTGAACGCTCTCTACTAGAGTCACACTGGCTCACCTTCGGGTGGGCCTTTCTGCGTTTATA

pHrpL.Rbs.λCl.T15

GCCGGATTATGTCCGCTGAGTGGGTCACGGTCCCGGATCAGTTCCCTTGCGAAGCTGACCGATGTTTTTGTGCCAAAAGCTGTTGTGGCAAAAAACGGTTTGCGCAAAGTTTTGTATTACAAAGAATTTCACATTTTAAAATATCTTTATAAATCAATCAGTTATTTCTATTTTTAAGCTGGCATGGTTATCGCTATAGGGCTTGTACTACTAGAAAAGAGGAGAAATACTAGATGAGCACAAAAAAGAAACCATTAACACAAGAGCAGCTTGAGGACGCACGTCGCCTTAAAGCAATTTATGAAAAAAAGAAAAATGAACTTGGCTTATCCCAGGAATCTGTCGCAGACAAGATGGGGATGGGGCAGTCAGGCGTTGGTGCTTTATTTAATGGCATCAATGCATTAAATGCTTATAACGCCGCATTGCTTGCAAAAATTCTCAAAGTTAGCGTTGAAGAATTTAGCCCTTCAATCGCCAGAGAAATCTACGAGATGTATGAAGCGGTTAGTATGCAGCCGTCACTTAGAAGTGAGTATGAGTACCCTGTTTTTTCTCATGTTCAGGCAGGGATGTTCTCACCTGAGCTTAGAACCTTTACCAAAGGTGATGCGGAGAGATGGGTAAGCACAACCAAAAAAGCCAGTGATTCTGCATTCTGGCTTGAGGTTGAAGGTAATTCCATGACCGCACCAACAGGCTCCAAGCCAAGCTTTCCTGACGGAATGTTAATTCTCGTTGACCCTGAGCAGGCTGTTGAGCCAGGTGATTTCTGCATAGCCAGACTTGGGGGTGATGAGTTTACCTTCAAGAAACTGATCAGGGATAGCGGTCAGGTGTTTTTACAACCACTAAACCCACAGTACCCAATGATCCCATGCAATGAGAGTTGTTCCGTTGTGGGGAAAGTTATCGCTAGTCAGTGGCCTGAAGAGACGTTTGGCTAATACTAGAGCCAGGCATCAAATAAAACGAAAGGCTCAGTCGAAAGACTGGGCCTTTCGTTTTATCTGTTGTTTGTCGGTGAACGCTCTCTACTAGAGTCACACTGGCTCACCTTCGGGTGGGCCTTTCTGCGTTTATA

**AND GATE (pSB4A5 plasmid, pSC101 replication origin)**

pHrpL.Rbs.GFP.T15-pBAD.Rbs.HrpS.T15-pRhaB.Rbs.HrpR.T15-pCON.Rbs.RhaS.Rbs.AraC.T15

GCCGGATTATGTCCGCTGAGTGGGTCACGGTCCCGGATCAGTTCCCTTGCGAAGCTGACCGATGTTTTTGTGCCAAAAGCTGTTGTGGCAAAAAACGGTTTGCGCAAAGTTTTGTATTACAAAGAATTTCACATTTTAAAATATCTTTATAAATCAATCAGTTATTTCTATTTTTAAGCTGGCATGGTTATCGCTATAGGGCTTGTACTACTAGAGATTAAAGAGGAGAAATACTAGATGCGTAAAGGAGAAGAACTTTTCACTGGAGTTGTCCCAATTCTTGTTGAATTAGATGGTGATGTTAATGGGCACAAATTTTCTGTCAGTGGAGAGGGTGAAGGTGATGCAACATACGGAAAACTTACCCTTAAATTTATTTGCACTACTGGAAAACTACCTGTTCCATGGCCAACACTTGTCACTACTTTCGGTTATGGTGTTCAATGCTTTGCGAGATACCCAGATCATATGAAACAGCATGACTTTTTCAAGAGTGCCATGCCCGAAGGTTATGTACAGGAAAGAACTATATTTTTCAAAGATGACGGGAACTACAAGACACGTGCTGAAGTCAAGTTTGAAGGTGATACCCTTGTTAATAGAATCGAGTTAAAAGGTATTGATTTTAAAGAAGATGGAAACATTCTTGGACACAAATTGGAATACAACTATAACTCACACAATGTATACATCATGGCAGACAAACAAAAGAATGGAATCAAAGTTAACTTCAAAATTAGACACAACATTGAAGATGGAAGCGTTCAACTAGCAGACCATTATCAACAAAATACTCCAATTGGCGATGGCCCTGTCCTTTTACCAGACAACCATTACCTGTCCACACAATCTGCCCTTTCGAAAGATCCCAACGAAAAGAGAGACCACATGGTCCTTCTTGAGTTTGTAACAGCTGCTGGGATTACACATGGCATGGATGAACTATACAAATAATAATACTAGAGCCAGGCATCAAATAAAACGAAAGGCTCAGTCGAAAGACTGGGCCTTTCGTTTTATCTGTTGTTTGTCGGTGAACGCTCTCTACTAGAGTCACACTGGCTCACCTTCGGGTGGGCCTTTCTGCGTTTATATACTAGAagaagaaaccaattgtccatattgcatcagacattgccgtcactgcgtcttttactggctcttctcgctaaccaaaccggtaaccccgcttattaaaagcattctgtaacaaagcgggaccaaagccatgacaaaaacgcgtaacaaaagtgtctataatcacggcagaaaagtccacattgattatttgcacggcgtcacactttgctatgccatagcatttttatccataagattagcggattctacctgacgctttttatcgcaactctctactgtttctccaT**A**TACTAGAGTCACACAGGACTACTAGATGACTATAATGATGAGTCTTGATGAAAGGTTTGAGGATGATCTGGACGAGGAGCGGGTTCCGAATCTGGGGATAGTTGCCGAAAGTATTTCGCAACTGGGTATCGACGTGCTGCTATCGGGTGAGACCGGCACGGGCAAAGACACGATTGCCCGACGGATTCATGAGATGTCAGGCCGCAAAGGGCGCCTGGTGGCGATGAATTGCGCGGCCATTCCGGAGTCCCTCGCCGAGAGCGAGTTATTCGGCGTGGTCAGCGGTGCCTACACCGGCGCTGATCGCTCCAGAGTCGGTTATGTCGAAGCGGCGCAGGGCGGCACGCTGTACCTGGATGAGATCGATAGCATGCCGCTGAGCCTGCAAGCCAAATTGCTGAGGGTGCTGGAAACCCGAGCGCTTGAACGGCTGGGTTCGACGTCGACGATCAAGCTGGATATCTGCGTGATCGCCTCCGCCCAATGCTCGCTGGACGACGCCGTCGAGCGGGGGCAGTTTCGTCGCGATCTGTATTTTCGCCTGAACGTCCTGACACTCAAGCTTCCTCCGCTACGTAACCAGTCTGATCGCATAGTTCCCCTGTTCACACGTTTTACGGCCGCCGCCGCGAGGGAGCTCGGTGTTCCCGTTCCCGATGTTTGCCCACTGCTGCACAAAGTGCTGCTGGGCCACGACTGGCCCGGCAATATCCGTGAGCTCAAGGCGGCAGCCAAACGCCATGTGCTGGGTTTCCCCTTGCTGGGCGCCGAGCCGCAGGGCGAAGAGCACTTGGCCTGTGGGCTCAAATCGCAATTGCGAGTGATCGAAAAAGCCCTGATTCAGGAGTCGCTCAAGCGCCACGACAATTGTGTGGATTCGGTAAGCCTGGAACTGGACGTGCCACGCCGTACGCTCTATCGACGCATCAAAGAATTGCAGATCTAATAACCAGGCATCAAATAAAACGAAAGGCTCAGTCGAAAGACTGGGCCTTTCGTTTTATCTGTTGTTTGTCGGTGAACGCTCTCTACTAGAGTCACACTGGCTCACCTTCGGGTGGGCCTTTCTGCGTTTATATAGACCTTTACGCCGCTGGAGCAGGAATGCGGTGAGCATCACATCACCACAATTCAGCAAATTGTGAACATCATCACGTTCATCTTTCCCTGGTTGCCAATGGCCCATTTTCCTGTCAGTAACGAGAAGGTCGCGAATTGAGGCGCTTTTTAGACTGGTCGT**A**TACTAGAGTCACACAGGAAACCTACTAGATGAGTACAGGCATCGATAAGGACGTCCGAGAGTGTTGGGGCGTAACTGCATTATCAGCGGGTCATCAAATTGCAATGAATAGCGCGTTTCTGGATATGGACTTGCTGTTGTGCGGGGAAACCGGCACCGGCAAGGACACACTGGCCAACCGCATTCACGAGTTGTCCAGCAGGTCGGGACCCTTTGTGGGCATGAACTGCGCCGCCATTCCCGAGTCGCTGGCAGAGAGCCAGTTATTCGGTGTGGTCAACGGTGCATTCACCGGCGTATGCCGGGCTCGCGAGGGCTACATAGAGGCCTCCAGTGGTGGCACCTTGTACCTGGATGAAATCGACAGCATGCCGTTGAGCCTGCAAGCCAAACTGCTGCGTGTGTTGGAGAGTCGAGGTATCGAGCGTCTGGGCTCGACCGAATTTATCCCGGTGGATCTGCGGATCATTGCCTCGGCCCAGCGGCCACTGGATGAACTGGTGGAACAAGGACTTTTCCGTCGCGACCTGTTTTTTCGGCTCAACGTGCTGACGCTTCACTTGCCAGCCTTGCGCAAACGTCGTGAACAGATCCTGCCATTGTTCGACCAGTTCACCCAGGGTATCGCTGCCGAGTTCGGACGTCCCGCTCCTGCGCTGGACAGCGGGCGTGTGCAGCTGCTGCTCAGCCACGACTGGCCGGGCAACATCCGCGAATTGAAGTCTGCGGCCAAGCGCTTCGTACTCGGCTTCCCCTTGCTGGGCGCCGACCCTGTGGAAGCGCTTGACCCTGCCACGGGGCTGCGCACGCAAATGCGCATCATCGAGAAAATGCTCATCCAGGATGCCTTGAAGCGGCACAGGCACAATTTCGACGCGGTGCTTCAGGAGTTGGAGTTGCCAAGACGCACCCTGTATCACCGCATGAAGGAACTGGGAGTTGCAGCGCCGATCGCTGCGACGGCCGGGGTCTAATAACCAGGCATCAAATAAAACGAAAGGCTCAGTCGAAAGACTGGGCCTTTCGTTTTATCTGTTGTTTGTCGGTGAACGCTCTCTACTAGAGTCACACTGGCTCACCTTCGGGTGGGCCTTTCTGCGTTTATATACTAGAtttacagctagctcagtcctaggtattatgctagcTACTAGAGGGAGCCCAAATGACCGTAttacatagtgtggatttttttccgtctggtaacgcgtccgtggcgatagaaccccggctcccgcaggcggattttcctgaacatcatcatgattttcatgaaattgtgattgtcgaacatggcacgggtattcatgtgtttaatgggcagccctataccatcaccggtggcacggtctgtttcgtacgcgatcatgatcggcatctgtatgaacataccgataatctgtgtctgaccaatgtgctgtatcgctcgccggatcgatttcagtttctcgccgggctgaatcagttgctgccacaagagctggatgggcagtatccgtctcactggcgcgttaaccacagcgtattgcagcaggtgcgacagctggttgcacagatggaacagcaggaaggggaaaatgatttaccctcgaccgccagtcgcgagatcttgtttatgcaattactgctcttgctgcgtaaaagcagtttgcaggagaacctggaaaacagcgcatcacgtctcaacttgcttctggcctggctggaggaccattttgccgatgaggtgaattgggatgccgtggcggatcaattttctctttcactgcgtacgctacatcggcagcttaagcagcaaacgggactgacgcctcagcgatacctgaaccgcctgcgactgatgaaagcccgacatctgctacgccacagcgaggccagcgttactgacatcgcctatcgctgtggattcagcgacagtaaccacttttcgacgctttttcgccgagagtttaactggtcaccgcgtgatattcgccagggacgggatggctttctgcaataaCGAGAGAGAGTTAACatggctgaagcgcaaaatgatcccctgctgccgggatactcgtttaacgcccatctggtggcgggtttaacgccgattgaggccaacggttatctcgatttttttatcgaccgaccgctgggaatgaaaggttatattctcaatctcaccattcgcggtcagggggtggtgaaaaatcagggacgagaatttgtctgccgaccgggtgatattttgctgttcccgccaggagagattcatcactacggtcgtcatccggaggctcgcgaatggtatcaccagtgggtttactttcgtccgcgcgcctactggcatgaatggcttaactggccgtcaatatttgccaatacgggtttctttcgcccggatgaagcgcaccagccgcatttcagcgacctgtttgggcaaatcattaacgccgggcaaggggaagggcgctattcggagctgctggcgataaatctgcttgagcaattgttactgcggcgcatggaagcgattaacgagtcgctccatccaccgatggataatcgggtacgcgaggcttgtcagtacatcagcgatcacctggcagacagcaattttgatatcgccagcgtcgcacagcatgtttgcttgtcgccgtcgcgtctgtcacatcttttccgccagcagttagggattagcgtcttaagctggcgcgaggaccaacgcattagtcaggcgaagctgcttttgagcactacccggatgcctatcgccaccgtcggtcgcaatgttggttttgacgatcaactctatttctcgcgagtatttaaaaaatgcaccggggccagcccgagcgagtttcgtgccggttgtgaagaaaaagtgaatgatgtagccgtcaagttgtcataaccaggcatcaaataaaacgaaaggctcagtcgaaagactgggcctttcgttttatctgttgtttgtcggtgaacgctctctactagagtcacactggctcaccttcgggtgggcctttctgcgtttata

**OR Gate I (pSB1C3, pMB1 replication origin)**

pCON.Rbs.RhaS.Rbs.AraC.T15-pBAD-pRHAB.RbsA.RFP.T15

tttacagctagctcagtcctaggtattatgctagcTACTAGAGGGAGCCCAAATGACCGTAttacatagtgtggatttttttccgtctggtaacgcgtccgtggcgatagaaccccggctcccgcaggcggattttcctgaacatcatcatgattttcatgaaattgtgattgtcgaacatggcacgggtattcatgtgtttaatgggcagccctataccatcaccggtggcacggtctgtttcgtacgcgatcatgatcggcatctgtatgaacataccgataatctgtgtctgaccaatgtgctgtatcgctcgccggatcgatttcagtttctcgccgggctgaatcagttgctgccacaagagctggatgggcagtatccgtctcactggcgcgttaaccacagcgtattgcagcaggtgcgacagctggttgcacagatggaacagcaggaaggggaaaatgatttaccctcgaccgccagtcgcgagatcttgtttatgcaattactgctcttgctgcgtaaaagcagtttgcaggagaacctggaaaacagcgcatcacgtctcaacttgcttctggcctggctggaggaccattttgccgatgaggtgaattgggatgccgtggcggatcaattttctctttcactgcgtacgctacatcggcagcttaagcagcaaacgggactgacgcctcagcgatacctgaaccgcctgcgactgatgaaagcccgacatctgctacgccacagcgaggccagcgttactgacatcgcctatcgctgtggattcagcgacagtaaccacttttcgacgctttttcgccgagagtttaactggtcaccgcgtgatattcgccagggacgggatggctttctgcaataaCGAGAGAGAGTTAACatggctgaagcgcaaaatgatcccctgctgccgggatactcgtttaacgcccatctggtggcgggtttaacgccgattgaggccaacggttatctcgatttttttatcgaccgaccgctgggaatgaaaggttatattctcaatctcaccattcgcggtcagggggtggtgaaaaatcagggacgagaatttgtctgccgaccgggtgatattttgctgttcccgccaggagagattcatcactacggtcgtcatccggaggctcgcgaatggtatcaccagtgggtttactttcgtccgcgcgcctactggcatgaatggcttaactggccgtcaatatttgccaatacgggtttctttcgcccggatgaagcgcaccagccgcatttcagcgacctgtttgggcaaatcattaacgccgggcaaggggaagggcgctattcggagctgctggcgataaatctgcttgagcaattgttactgcggcgcatggaagcgattaacgagtcgctccatccaccgatggataatcgggtacgcgaggcttgtcagtacatcagcgatcacctggcagacagcaattttgatatcgccagcgtcgcacagcatgtttgcttgtcgccgtcgcgtctgtcacatcttttccgccagcagttagggattagcgtcttaagctggcgcgaggaccaacgcattagtcaggcgaagctgcttttgagcactacccggatgcctatcgccaccgtcggtcgcaatgttggttttgacgatcaactctatttctcgcgagtatttaaaaaatgcaccggggccagcccgagcgagtttcgtgccggttgtgaagaaaaagtgaatgatgtagccgtcaagttgtcataaccaggcatcaaataaaacgaaaggctcagtcgaaagactgggcctttcgttttatctgttgtttgtcggtgaacgctctctactagagtcacactggctcaccttcgggtgggcctttctgcgtttataTACTAGAGagaagaaaccaattgtccatattgcatcagacattgccgtcactgcgtcttttactggctcttctcgctaaccaaaccggtaaccccgcttattaaaagcattctgtaacaaagcgggaccaaagccatgacaaaaacgcgtaacaaaagtgtctataatcacggcagaaaagtccacattgattatttgcacggcgtcacactttgctatgccatagcatttttatccataagattagcggattctacctgacgctttttatcgcaactctctactgtttctccaT**A**TACTAGAGAGACCTTTACGCCGCTGGAGCAGGAATGCGGTGAGCATCACATCACCACAATTCAGCAAATTGTGAACATCATCACGTTCATCTTTCCCTGGTTGCCAATGGCCCATTTTCCTGTCAGTAACGAGAAGGTCGCGAATTGAGGCGCTTTTTAGACTGGTCGT**A**TACTAGAGAAAGAGGAGAAAATGGCTTCCTCCGAAGACGTTATCAAAGAGTTCATGCGTTTCAAAGTTCGTATGGAAGGTTCCGTTAACGGTCACGAGTTCGAAATCGAAGGTGAAGGTGAAGGTCGTCCGTACGAAGGTACCCAGACCGCTAAACTGAAAGTTACCAAAGGTGGTCCGCTGCCGTTCGCTTGGGACATCCTGTCCCCGCAGTTCCAGTACGGTTCCAAAGCTTACGTTAAACACCCGGCTGACATCCCGGACTACCTGAAACTGTCCTTCCCGGAAGGTTTCAAATGGGAACGTGTTATGAACTTCGAAGACGGTGGTGTTGTTACCGTTACCCAGGACTCCTCCCTGCAAGACGGTGAGTTCATCTACAAAGTTAAACTGCGTGGTACCAACTTCCCGTCCGACGGTCCGGTTATGCAGAAAAAAACCATGGGTTGGGAAGCTTCCACCGAACGTATGTACCCGGAAGACGGTGCTCTGAAAGGTGAAATCAAAATGCGTCTGAAACTGAAAGACGGTGGTCACTACGACGCTGAAGTTAAAACCACCTACATGGCTAAAAAACCGGTTCAGCTGCCGGGTGCTTACAAAACCGACATCAAACTGGACATCACCTCCCACAACGAAGACTACACCATCGTTGAACAGTACGAACGTGCTGAAGGTCGTCACTCCACCGGTGCTTAATAATACTAGAGCCAGGCATCAAATAAAACGAAAGGCTCAGTCGAAAGACTGGGCCTTTCGTTTTATCTGTTGTTTGTCGGTGAACGCTCTCTACTAGAGTCACACTGGCTCACCTTCGGGTGGGCCTTTCTGCGTTTATA

**OR Gate II (pSB1C3, pMB1 replication origin)**

pCON.Rbs.RhaS.Rbs.AraC.T15-pRHAB-pBAD.RbsA.RFP.T15

tttacagctagctcagtcctaggtattatgctagcTACTAGAGGGAGCCCAAATGACCGTAttacatagtgtggatttttttccgtctggtaacgcgtccgtggcgatagaaccccggctcccgcaggcggattttcctgaacatcatcatgattttcatgaaattgtgattgtcgaacatggcacgggtattcatgtgtttaatgggcagccctataccatcaccggtggcacggtctgtttcgtacgcgatcatgatcggcatctgtatgaacataccgataatctgtgtctgaccaatgtgctgtatcgctcgccggatcgatttcagtttctcgccgggctgaatcagttgctgccacaagagctggatgggcagtatccgtctcactggcgcgttaaccacagcgtattgcagcaggtgcgacagctggttgcacagatggaacagcaggaaggggaaaatgatttaccctcgaccgccagtcgcgagatcttgtttatgcaattactgctcttgctgcgtaaaagcagtttgcaggagaacctggaaaacagcgcatcacgtctcaacttgcttctggcctggctggaggaccattttgccgatgaggtgaattgggatgccgtggcggatcaattttctctttcactgcgtacgctacatcggcagcttaagcagcaaacgggactgacgcctcagcgatacctgaaccgcctgcgactgatgaaagcccgacatctgctacgccacagcgaggccagcgttactgacatcgcctatcgctgtggattcagcgacagtaaccacttttcgacgctttttcgccgagagtttaactggtcaccgcgtgatattcgccagggacgggatggctttctgcaataaCGAGAGAGAGTTAACatggctgaagcgcaaaatgatcccctgctgccgggatactcgtttaacgcccatctggtggcgggtttaacgccgattgaggccaacggttatctcgatttttttatcgaccgaccgctgggaatgaaaggttatattctcaatctcaccattcgcggtcagggggtggtgaaaaatcagggacgagaatttgtctgccgaccgggtgatattttgctgttcccgccaggagagattcatcactacggtcgtcatccggaggctcgcgaatggtatcaccagtgggtttactttcgtccgcgcgcctactggcatgaatggcttaactggccgtcaatatttgccaatacgggtttctttcgcccggatgaagcgcaccagccgcatttcagcgacctgtttgggcaaatcattaacgccgggcaaggggaagggcgctattcggagctgctggcgataaatctgcttgagcaattgttactgcggcgcatggaagcgattaacgagtcgctccatccaccgatggataatcgggtacgcgaggcttgtcagtacatcagcgatcacctggcagacagcaattttgatatcgccagcgtcgcacagcatgtttgcttgtcgccgtcgcgtctgtcacatcttttccgccagcagttagggattagcgtcttaagctggcgcgaggaccaacgcattagtcaggcgaagctgcttttgagcactacccggatgcctatcgccaccgtcggtcgcaatgttggttttgacgatcaactctatttctcgcgagtatttaaaaaatgcaccggggccagcccgagcgagtttcgtgccggttgtgaagaaaaagtgaatgatgtagccgtcaagttgtcataaccaggcatcaaataaaacgaaaggctcagtcgaaagactgggcctttcgttttatctgttgtttgtcggtgaacgctctctactagagtcacactggctcaccttcgggtgggcctttctgcgtttataTACTAGAGAGACCTTTACGCCGCTGGAGCAGGAATGCGGTGAGCATCACATCACCACAATTCAGCAAATTGTGAACATCATCACGTTCATCTTTCCCTGGTTGCCAATGGCCCATTTTCCTGTCAGTAACGAGAAGGTCGCGAATTGAGGCGCTTTTTAGACTGGTCGT**A**TACTAGAGagaagaaaccaattgtccatattgcatcagacattgccgtcactgcgtcttttactggctcttctcgctaaccaaaccggtaaccccgcttattaaaagcattctgtaacaaagcgggaccaaagccatgacaaaaacgcgtaacaaaagtgtctataatcacggcagaaaagtccacattgattatttgcacggcgtcacactttgctatgccatagcatttttatccataagattagcggattctacctgacgctttttatcgcaactctctactgtttctccaT**A**AAAGAGGAGAAAATGGCTTCCTCCGAAGACGTTATCAAAGAGTTCATGCGTTTCAAAGTTCGTATGGAAGGTTCCGTTAACGGTCACGAGTTCGAAATCGAAGGTGAAGGTGAAGGTCGTCCGTACGAAGGTACCCAGACCGCTAAACTGAAAGTTACCAAAGGTGGTCCGCTGCCGTTCGCTTGGGACATCCTGTCCCCGCAGTTCCAGTACGGTTCCAAAGCTTACGTTAAACACCCGGCTGACATCCCGGACTACCTGAAACTGTCCTTCCCGGAAGGTTTCAAATGGGAACGTGTTATGAACTTCGAAGACGGTGGTGTTGTTACCGTTACCCAGGACTCCTCCCTGCAAGACGGTGAGTTCATCTACAAAGTTAAACTGCGTGGTACCAACTTCCCGTCCGACGGTCCGGTTATGCAGAAAAAAACCATGGGTTGGGAAGCTTCCACCGAACGTATGTACCCGGAAGACGGTGCTCTGAAAGGTGAAATCAAAATGCGTCTGAAACTGAAAGACGGTGGTCACTACGACGCTGAAGTTAAAACCACCTACATGGCTAAAAAACCGGTTCAGCTGCCGGGTGCTTACAAAACCGACATCAAACTGGACATCACCTCCCACAACGAAGACTACACCATCGTTGAACAGTACGAACGTGCTGAAGGTCGTCACTCCACCGGTGCTTAATAATACTAGAGCCAGGCATCAAATAAAACGAAAGGCTCAGTCGAAAGACTGGGCCTTTCGTTTTATCTGTTGTTTGTCGGTGAACGCTCTCTACTAGAGTCACACTGGCTCACCTTCGGGTGGGCCTTTCTGCGTTTATA

**OR Gate III (pSB1C3, pMB1 replication origin)**

pCON.Rbs.RhaS.Rbs.AraC.T15-pBAD.RbsA.RFP.T15-pRHAB.RbsA.RFP.T15

tttacagctagctcagtcctaggtattatgctagcTACTAGAGGGAGCCCAAATGACCGTAttacatagtgtggatttttttccgtctggtaacgcgtccgtggcgatagaaccccggctcccgcaggcggattttcctgaacatcatcatgattttcatgaaattgtgattgtcgaacatggcacgggtattcatgtgtttaatgggcagccctataccatcaccggtggcacggtctgtttcgtacgcgatcatgatcggcatctgtatgaacataccgataatctgtgtctgaccaatgtgctgtatcgctcgccggatcgatttcagtttctcgccgggctgaatcagttgctgccacaagagctggatgggcagtatccgtctcactggcgcgttaaccacagcgtattgcagcaggtgcgacagctggttgcacagatggaacagcaggaaggggaaaatgatttaccctcgaccgccagtcgcgagatcttgtttatgcaattactgctcttgctgcgtaaaagcagtttgcaggagaacctggaaaacagcgcatcacgtctcaacttgcttctggcctggctggaggaccattttgccgatgaggtgaattgggatgccgtggcggatcaattttctctttcactgcgtacgctacatcggcagcttaagcagcaaacgggactgacgcctcagcgatacctgaaccgcctgcgactgatgaaagcccgacatctgctacgccacagcgaggccagcgttactgacatcgcctatcgctgtggattcagcgacagtaaccacttttcgacgctttttcgccgagagtttaactggtcaccgcgtgatattcgccagggacgggatggctttctgcaataaCGAGAGAGAGTTAACatggctgaagcgcaaaatgatcccctgctgccgggatactcgtttaacgcccatctggtggcgggtttaacgccgattgaggccaacggttatctcgatttttttatcgaccgaccgctgggaatgaaaggttatattctcaatctcaccattcgcggtcagggggtggtgaaaaatcagggacgagaatttgtctgccgaccgggtgatattttgctgttcccgccaggagagattcatcactacggtcgtcatccggaggctcgcgaatggtatcaccagtgggtttactttcgtccgcgcgcctactggcatgaatggcttaactggccgtcaatatttgccaatacgggtttctttcgcccggatgaagcgcaccagccgcatttcagcgacctgtttgggcaaatcattaacgccgggcaaggggaagggcgctattcggagctgctggcgataaatctgcttgagcaattgttactgcggcgcatggaagcgattaacgagtcgctccatccaccgatggataatcgggtacgcgaggcttgtcagtacatcagcgatcacctggcagacagcaattttgatatcgccagcgtcgcacagcatgtttgcttgtcgccgtcgcgtctgtcacatcttttccgccagcagttagggattagcgtcttaagctggcgcgaggaccaacgcattagtcaggcgaagctgcttttgagcactacccggatgcctatcgccaccgtcggtcgcaatgttggttttgacgatcaactctatttctcgcgagtatttaaaaaatgcaccggggccagcccgagcgagtttcgtgccggttgtgaagaaaaagtgaatgatgtagccgtcaagttgtcataaccaggcatcaaataaaacgaaaggctcagtcgaaagactgggcctttcgttttatctgttgtttgtcggtgaacgctctctactagagtcacactggctcaccttcgggtgggcctttctgcgtttataTACTAGAGagaagaaaccaattgtccatattgcatcagacattgccgtcactgcgtcttttactggctcttctcgctaaccaaaccggtaaccccgcttattaaaagcattctgtaacaaagcgggaccaaagccatgacaaaaacgcgtaacaaaagtgtctataatcacggcagaaaagtccacattgattatttgcacggcgtcacactttgctatgccatagcatttttatccataagattagcggattctacctgacgctttttatcgcaactctctactgtttctccaT**A**TACTAGAGAAAGAGGAGAAAATGGCTTCCTCCGAAGACGTTATCAAAGAGTTCATGCGTTTCAAAGTTCGTATGGAAGGTTCCGTTAACGGTCACGAGTTCGAAATCGAAGGTGAAGGTGAAGGTCGTCCGTACGAAGGTACCCAGACCGCTAAACTGAAAGTTACCAAAGGTGGTCCGCTGCCGTTCGCTTGGGACATCCTGTCCCCGCAGTTCCAGTACGGTTCCAAAGCTTACGTTAAACACCCGGCTGACATCCCGGACTACCTGAAACTGTCCTTCCCGGAAGGTTTCAAATGGGAACGTGTTATGAACTTCGAAGACGGTGGTGTTGTTACCGTTACCCAGGACTCCTCCCTGCAAGACGGTGAGTTCATCTACAAAGTTAAACTGCGTGGTACCAACTTCCCGTCCGACGGTCCGGTTATGCAGAAAAAAACCATGGGTTGGGAAGCTTCCACCGAACGTATGTACCCGGAAGACGGTGCTCTGAAAGGTGAAATCAAAATGCGTCTGAAACTGAAAGACGGTGGTCACTACGACGCTGAAGTTAAAACCACCTACATGGCTAAAAAACCGGTTCAGCTGCCGGGTGCTTACAAAACCGACATCAAACTGGACATCACCTCCCACAACGAAGACTACACCATCGTTGAACAGTACGAACGTGCTGAAGGTCGTCACTCCACCGGTGCTTAATAATACTAGAGCCAGGCATCAAATAAAACGAAAGGCTCAGTCGAAAGACTGGGCCTTTCGTTTTATCTGTTGTTTGTCGGTGAACGCTCTCTACTAGAGTCACACTGGCTCACCTTCGGGTGGGCCTTTCTGCGTTTATATACTAGAGAGACCTTTACGCCGCTGGAGCAGGAATGCGGTGAGCATCACATCACCACAATTCAGCAAATTGTGAACATCATCACGTTCATCTTTCCCTGGTTGCCAATGGCCCATTTTCCTGTCAGTAACGAGAAGGTCGCGAATTGAGGCGCTTTTTAGACTGGTCGT**A**TACTAGAGAAAGAGGAGAAAATGGCTTCCTCCGAAGACGTTATCAAAGAGTTCATGCGTTTCAAAGTTCGTATGGAAGGTTCCGTTAACGGTCACGAGTTCGAAATCGAAGGTGAAGGTGAAGGTCGTCCGTACGAAGGTACCCAGACCGCTAAACTGAAAGTTACCAAAGGTGGTCCGCTGCCGTTCGCTTGGGACATCCTGTCCCCGCAGTTCCAGTACGGTTCCAAAGCTTACGTTAAACACCCGGCTGACATCCCGGACTACCTGAAACTGTCCTTCCCGGAAGGTTTCAAATGGGAACGTGTTATGAACTTCGAAGACGGTGGTGTTGTTACCGTTACCCAGGACTCCTCCCTGCAAGACGGTGAGTTCATCTACAAAGTTAAACTGCGTGGTACCAACTTCCCGTCCGACGGTCCGGTTATGCAGAAAAAAACCATGGGTTGGGAAGCTTCCACCGAACGTATGTACCCGGAAGACGGTGCTCTGAAAGGTGAAATCAAAATGCGTCTGAAACTGAAAGACGGTGGTCACTACGACGCTGAAGTTAAAACCACCTACATGGCTAAAAAACCGGTTCAGCTGCCGGGTGCTTACAAAACCGACATCAAACTGGACATCACCTCCCACAACGAAGACTACACCATCGTTGAACAGTACGAACGTGCTGAAGGTCGTCACTCCACCGGTGCTTAATAATACTAGAGCCAGGCATCAAATAAAACGAAAGGCTCAGTCGAAAGACTGGGCCTTTCGTTTTATCTGTTGTTTGTCGGTGAACGCTCTCTACTAGAGTCACACTGGCTCACCTTCGGGTGGGCCTTTCTGCGTTTATA

**NIMPLY GATE** **(pSB1C3, pMB1 replication origin)**

*TATCACCGGGCGTGCTGATA palindromic λCl binding sites

pCON.RbsRhaS.Rbs.AraC.T15.pBAD.pRHAB_Cl2B_.RbsA.RFP.T15

tttacagctagctcagtcctaggtattatgctagcTACTAGAGGGAGCCCAAATGACCGTAttacatagtgtggatttttttccgtctggtaacgcgtccgtggcgatagaaccccggctcccgcaggcggattttcctgaacatcatcatgattttcatgaaattgtgattgtcgaacatggcacgggtattcatgtgtttaatgggcagccctataccatcaccggtggcacggtctgtttcgtacgcgatcatgatcggcatctgtatgaacataccgataatctgtgtctgaccaatgtgctgtatcgctcgccggatcgatttcagtttctcgccgggctgaatcagttgctgccacaagagctggatgggcagtatccgtctcactggcgcgttaaccacagcgtattgcagcaggtgcgacagctggttgcacagatggaacagcaggaaggggaaaatgatttaccctcgaccgccagtcgcgagatcttgtttatgcaattactgctcttgctgcgtaaaagcagtttgcaggagaacctggaaaacagcgcatcacgtctcaacttgcttctggcctggctggaggaccattttgccgatgaggtgaattgggatgccgtggcggatcaattttctctttcactgcgtacgctacatcggcagcttaagcagcaaacgggactgacgcctcagcgatacctgaaccgcctgcgactgatgaaagcccgacatctgctacgccacagcgaggccagcgttactgacatcgcctatcgctgtggattcagcgacagtaaccacttttcgacgctttttcgccgagagtttaactggtcaccgcgtgatattcgccagggacgggatggctttctgcaataaCGAGAGAGAGTTAACatggctgaagcgcaaaatgatcccctgctgccgggatactcgtttaacgcccatctggtggcgggtttaacgccgattgaggccaacggttatctcgatttttttatcgaccgaccgctgggaatgaaaggttatattctcaatctcaccattcgcggtcagggggtggtgaaaaatcagggacgagaatttgtctgccgaccgggtgatattttgctgttcccgccaggagagattcatcactacggtcgtcatccggaggctcgcgaatggtatcaccagtgggtttactttcgtccgcgcgcctactggcatgaatggcttaactggccgtcaatatttgccaatacgggtttctttcgcccggatgaagcgcaccagccgcatttcagcgacctgtttgggcaaatcattaacgccgggcaaggggaagggcgctattcggagctgctggcgataaatctgcttgagcaattgttactgcggcgcatggaagcgattaacgagtcgctccatccaccgatggataatcgggtacgcgaggcttgtcagtacatcagcgatcacctggcagacagcaattttgatatcgccagcgtcgcacagcatgtttgcttgtcgccgtcgcgtctgtcacatcttttccgccagcagttagggattagcgtcttaagctggcgcgaggaccaacgcattagtcaggcgaagctgcttttgagcactacccggatgcctatcgccaccgtcggtcgcaatgttggttttgacgatcaactctatttctcgcgagtatttaaaaaatgcaccggggccagcccgagcgagtttcgtgccggttgtgaagaaaaagtgaatgatgtagccgtcaagttgtcataaccaggcatcaaataaaacgaaaggctcagtcgaaagactgggcctttcgttttatctgttgtttgtcggtgaacgctctctactagagtcacactggctcaccttcgggtgggcctttctgcgtttataTACTAGAGagaagaaaccaattgtccatattgcatcagacattgccgtcactgcgtcttttactggctcttctcgctaaccaaaccggtaaccccgcttattaaaagcattctgtaacaaagcgggaccaaagccatgacaaaaacgcgtaacaaaagtgtctataatcacggcagaaaagtccacattgattatttgcacggcgtcacactttgctatgccatagcatttttatccataagattagcggattctacctgacgctttttatcgcaactctctactgtttctccaT**A**TACTAGAAAAGAGGAGAAATACTAGATGAGCACAAAAAAGAAACCATTAACACAAGAGCAGCTTGAGGACGCACGTCGCCTTAAAGCAATTTATGAAAAAAAGAAAAATGAACTTGGCTTATCCCAGGAATCTGTCGCAGACAAGATGGGGATGGGGCAGTCAGGCGTTGGTGCTTTATTTAATGGCATCAATGCATTAAATGCTTATAACGCCGCATTGCTTGCAAAAATTCTCAAAGTTAGCGTTGAAGAATTTAGCCCTTCAATCGCCAGAGAAATCTACGAGATGTATGAAGCGGTTAGTATGCAGCCGTCACTTAGAAGTGAGTATGAGTACCCTGTTTTTTCTCATGTTCAGGCAGGGATGTTCTCACCTGAGCTTAGAACCTTTACCAAAGGTGATGCGGAGAGATGGGTAAGCACAACCAAAAAAGCCAGTGATTCTGCATTCTGGCTTGAGGTTGAAGGTAATTCCATGACCGCACCAACAGGCTCCAAGCCAAGCTTTCCTGACGGAATGTTAATTCTCGTTGACCCTGAGCAGGCTGTTGAGCCAGGTGATTTCTGCATAGCCAGACTTGGGGGTGATGAGTTTACCTTCAAGAAACTGATCAGGGATAGCGGTCAGGTGTTTTTACAACCACTAAACCCACAGTACCCAATGATCCCATGCAATGAGAGTTGTTCCGTTGTGGGGAAAGTTATCGCTAGTCAGTGGCCTGAAGAGACGTTTGGCTAATACTAGAGCCAGGCATCAAATAAAACGAAAGGCTCAGTCGAAAGACTGGGCCTTTCGTTTTATCTGTTGTTTGTCGGTGAACGCTCTCTACTAGAGTCACACTGGCTCACCTTCGGGTGGGCCTTTCTGCGTTTATAagaagaaaccaattgtccatattgcatcagacattgccgtcactgcgtcttttactggctcttctcgctaaccaaaccggtaaccccgcttattaaaagcattctgtaacaaagcgggaccaaagccatgacaaaaacgcgtaacaaaagtgtctataatcacggcagaaaagtccacattgattatttgcacggcgtcacactttgctatgccatagcatttttatccataagattagcggattctacctgacgctttttatcgcaactctctactgtttctccaT**A**TACTAGAGAGACCTTTACGCCGCTGGAGCAGGAATGCGGTGAGCATCACATCACCACAATTCAGCAAATTGTGAACATCATCACGTTCATCTTTCCCTGGTTGCCAATGGCCCATTTTCCTGTCAGTAACGAGAAGGTCGCGAATTGAGGCGCTTTTTAGACTGGTCGT**A**TATCACCGGGCGTGCTGATACAGTCATACTAGAAAAGAGGAGAAAATGGCTTCCTCCGAAGACGTTATCAAAGAGTTCATGCGTTTCAAAGTTCGTATGGAAGGTTCCGTTAACGGTCACGAGTTCGAAATCGAAGGTGAAGGTGAAGGTCGTCCGTACGAAGGTACCCAGACCGCTAAACTGAAAGTTACCAAAGGTGGTCCGCTGCCGTTCGCTTGGGACATCCTGTCCCCGCAGTTCCAGTACGGTTCCAAAGCTTACGTTAAACACCCGGCTGACATCCCGGACTACCTGAAACTGTCCTTCCCGGAAGGTTTCAAATGGGAACGTGTTATGAACTTCGAAGACGGTGGTGTTGTTACCGTTACCCAGGACTCCTCCCTGCAAGACGGTGAGTTCATCTACAAAGTTAAACTGCGTGGTACCAACTTCCCGTCCGACGGTCCGGTTATGCAGAAAAAAACCATGGGTTGGGAAGCTTCCACCGAACGTATGTACCCGGAAGACGGTGCTCTGAAAGGTGAAATCAAAATGCGTCTGAAACTGAAAGACGGTGGTCACTACGACGCTGAAGTTAAAACCACCTACATGGCTAAAAAACCGGTTCAGCTGCCGGGTGCTTACAAAACCGACATCAAACTGGACATCACCTCCCACAACGAAGACTACACCATCGTTGAACAGTACGAACGTGCTGAAGGTCGTCACTCCACCGGTGCTTAATAATACTAGAGCCAGGCATCAAATAAAACGAAAGGCTCAGTCGAAAGACTGGGCCTTTCGTTTTATCTGTTGTTTGTCGGTGAACGCTCTCTACTAGAGTCACACTGGCTCACCTTCGGGTGGGCCTTTCTGCGTTTATA

pCON.RbsRhaS.Rbs.AraC.T15.pBAD.pRHAB_Cl2B_.RbsB.RFP.T15

tttacagctagctcagtcctaggtattatgctagcTACTAGAGGGAGCCCAAATGACCGTAttacatagtgtggatttttttccgtctggtaacgcgtccgtggcgatagaaccccggctcccgcaggcggattttcctgaacatcatcatgattttcatgaaattgtgattgtcgaacatggcacgggtattcatgtgtttaatgggcagccctataccatcaccggtggcacggtctgtttcgtacgcgatcatgatcggcatctgtatgaacataccgataatctgtgtctgaccaatgtgctgtatcgctcgccggatcgatttcagtttctcgccgggctgaatcagttgctgccacaagagctggatgggcagtatccgtctcactggcgcgttaaccacagcgtattgcagcaggtgcgacagctggttgcacagatggaacagcaggaaggggaaaatgatttaccctcgaccgccagtcgcgagatcttgtttatgcaattactgctcttgctgcgtaaaagcagtttgcaggagaacctggaaaacagcgcatcacgtctcaacttgcttctggcctggctggaggaccattttgccgatgaggtgaattgggatgccgtggcggatcaattttctctttcactgcgtacgctacatcggcagcttaagcagcaaacgggactgacgcctcagcgatacctgaaccgcctgcgactgatgaaagcccgacatctgctacgccacagcgaggccagcgttactgacatcgcctatcgctgtggattcagcgacagtaaccacttttcgacgctttttcgccgagagtttaactggtcaccgcgtgatattcgccagggacgggatggctttctgcaataaCGAGAGAGAGTTAACatggctgaagcgcaaaatgatcccctgctgccgggatactcgtttaacgcccatctggtggcgggtttaacgccgattgaggccaacggttatctcgatttttttatcgaccgaccgctgggaatgaaaggttatattctcaatctcaccattcgcggtcagggggtggtgaaaaatcagggacgagaatttgtctgccgaccgggtgatattttgctgttcccgccaggagagattcatcactacggtcgtcatccggaggctcgcgaatggtatcaccagtgggtttactttcgtccgcgcgcctactggcatgaatggcttaactggccgtcaatatttgccaatacgggtttctttcgcccggatgaagcgcaccagccgcatttcagcgacctgtttgggcaaatcattaacgccgggcaaggggaagggcgctattcggagctgctggcgataaatctgcttgagcaattgttactgcggcgcatggaagcgattaacgagtcgctccatccaccgatggataatcgggtacgcgaggcttgtcagtacatcagcgatcacctggcagacagcaattttgatatcgccagcgtcgcacagcatgtttgcttgtcgccgtcgcgtctgtcacatcttttccgccagcagttagggattagcgtcttaagctggcgcgaggaccaacgcattagtcaggcgaagctgcttttgagcactacccggatgcctatcgccaccgtcggtcgcaatgttggttttgacgatcaactctatttctcgcgagtatttaaaaaatgcaccggggccagcccgagcgagtttcgtgccggttgtgaagaaaaagtgaatgatgtagccgtcaagttgtcataaccaggcatcaaataaaacgaaaggctcagtcgaaagactgggcctttcgttttatctgttgtttgtcggtgaacgctctctactagagtcacactggctcaccttcgggtgggcctttctgcgtttataTACTAGAGagaagaaaccaattgtccatattgcatcagacattgccgtcactgcgtcttttactggctcttctcgctaaccaaaccggtaaccccgcttattaaaagcattctgtaacaaagcgggaccaaagccatgacaaaaacgcgtaacaaaagtgtctataatcacggcagaaaagtccacattgattatttgcacggcgtcacactttgctatgccatagcatttttatccataagattagcggattctacctgacgctttttatcgcaactctctactgtttctccaT**A**TACTAGAAAAGAGGAGAAATACTAGATGAGCACAAAAAAGAAACCATTAACACAAGAGCAGCTTGAGGACGCACGTCGCCTTAAAGCAATTTATGAAAAAAAGAAAAATGAACTTGGCTTATCCCAGGAATCTGTCGCAGACAAGATGGGGATGGGGCAGTCAGGCGTTGGTGCTTTATTTAATGGCATCAATGCATTAAATGCTTATAACGCCGCATTGCTTGCAAAAATTCTCAAAGTTAGCGTTGAAGAATTTAGCCCTTCAATCGCCAGAGAAATCTACGAGATGTATGAAGCGGTTAGTATGCAGCCGTCACTTAGAAGTGAGTATGAGTACCCTGTTTTTTCTCATGTTCAGGCAGGGATGTTCTCACCTGAGCTTAGAACCTTTACCAAAGGTGATGCGGAGAGATGGGTAAGCACAACCAAAAAAGCCAGTGATTCTGCATTCTGGCTTGAGGTTGAAGGTAATTCCATGACCGCACCAACAGGCTCCAAGCCAAGCTTTCCTGACGGAATGTTAATTCTCGTTGACCCTGAGCAGGCTGTTGAGCCAGGTGATTTCTGCATAGCCAGACTTGGGGGTGATGAGTTTACCTTCAAGAAACTGATCAGGGATAGCGGTCAGGTGTTTTTACAACCACTAAACCCACAGTACCCAATGATCCCATGCAATGAGAGTTGTTCCGTTGTGGGGAAAGTTATCGCTAGTCAGTGGCCTGAAGAGACGTTTGGCTAATACTAGAGCCAGGCATCAAATAAAACGAAAGGCTCAGTCGAAAGACTGGGCCTTTCGTTTTATCTGTTGTTTGTCGGTGAACGCTCTCTACTAGAGTCACACTGGCTCACCTTCGGGTGGGCCTTTCTGCGTTTATAagaagaaaccaattgtccatattgcatcagacattgccgtcactgcgtcttttactggctcttctcgctaaccaaaccggtaaccccgcttattaaaagcattctgtaacaaagcgggaccaaagccatgacaaaaacgcgtaacaaaagtgtctataatcacggcagaaaagtccacattgattatttgcacggcgtcacactttgctatgccatagcatttttatccataagattagcggattctacctgacgctttttatcgcaactctctactgtttctccaT**A**TACTAGAGAGACCTTTACGCCGCTGGAGCAGGAATGCGGTGAGCATCACATCACCACAATTCAGCAAATTGTGAACATCATCACGTTCATCTTTCCCTGGTTGCCAATGGCCCATTTTCCTGTCAGTAACGAGAAGGTCGCGAATTGAGGCGCTTTTTAGACTGGTCGT**A**TATCACCGGGCGTGCTGATACAGTCATACTAGATCACACAGGAAAGTACTAGATGGCTTCCTCCGAAGACGTTATCAAAGAGTTCATGCGTTTCAAAGTTCGTATGGAAGGTTCCGTTAACGGTCACGAGTTCGAAATCGAAGGTGAAGGTGAAGGTCGTCCGTACGAAGGTACCCAGACCGCTAAACTGAAAGTTACCAAAGGTGGTCCGCTGCCGTTCGCTTGGGACATCCTGTCCCCGCAGTTCCAGTACGGTTCCAAAGCTTACGTTAAACACCCGGCTGACATCCCGGACTACCTGAAACTGTCCTTCCCGGAAGGTTTCAAATGGGAACGTGTTATGAACTTCGAAGACGGTGGTGTTGTTACCGTTACCCAGGACTCCTCCCTGCAAGACGGTGAGTTCATCTACAAAGTTAAACTGCGTGGTACCAACTTCCCGTCCGACGGTCCGGTTATGCAGAAAAAAACCATGGGTTGGGAAGCTTCCACCGAACGTATGTACCCGGAAGACGGTGCTCTGAAAGGTGAAATCAAAATGCGTCTGAAACTGAAAGACGGTGGTCACTACGACGCTGAAGTTAAAACCACCTACATGGCTAAAAAACCGGTTCAGCTGCCGGGTGCTTACAAAACCGACATCAAACTGGACATCACCTCCCACAACGAAGACTACACCATCGTTGAACAGTACGAACGTGCTGAAGGTCGTCACTCCACCGGTGCTTAATAATACTAGAGCCAGGCATCAAATAAAACGAAAGGCTCAGTCGAAAGACTGGGCCTTTCGTTTTATCTGTTGTTTGTCGGTGAACGCTCTCTACTAGAGTCACACTGGCTCACCTTCGGGTGGGCCTTTCTGCGTTTATA

**XOR GATE (AND GATE in pSB4A5 & OR-NOT GATE in pSB1C3)**

pBAD.Rbs.HrpS.T15-pRhaB.Rbs.HrpR.T15-pCON.Rbs.RhaS.Rbs.AraC.T15 [pSB4A5]

agaagaaaccaattgtccatattgcatcagacattgccgtcactgcgtcttttactggctcttctcgctaaccaaaccggtaaccccgcttattaaaagcattctgtaacaaagcgggaccaaagccatgacaaaaacgcgtaacaaaagtgtctataatcacggcagaaaagtccacattgattatttgcacggcgtcacactttgctatgccatagcatttttatccataagattagcggattctacctgacgctttttatcgcaactctctactgtttctccaT**A**TACTAGAGTCACACAGGACTACTAGATGACTATAATGATGAGTCTTGATGAAAGGTTTGAGGATGATCTGGACGAGGAGCGGGTTCCGAATCTGGGGATAGTTGCCGAAAGTATTTCGCAACTGGGTATCGACGTGCTGCTATCGGGTGAGACCGGCACGGGCAAAGACACGATTGCCCGACGGATTCATGAGATGTCAGGCCGCAAAGGGCGCCTGGTGGCGATGAATTGCGCGGCCATTCCGGAGTCCCTCGCCGAGAGCGAGTTATTCGGCGTGGTCAGCGGTGCCTACACCGGCGCTGATCGCTCCAGAGTCGGTTATGTCGAAGCGGCGCAGGGCGGCACGCTGTACCTGGATGAGATCGATAGCATGCCGCTGAGCCTGCAAGCCAAATTGCTGAGGGTGCTGGAAACCCGAGCGCTTGAACGGCTGGGTTCGACGTCGACGATCAAGCTGGATATCTGCGTGATCGCCTCCGCCCAATGCTCGCTGGACGACGCCGTCGAGCGGGGGCAGTTTCGTCGCGATCTGTATTTTCGCCTGAACGTCCTGACACTCAAGCTTCCTCCGCTACGTAACCAGTCTGATCGCATAGTTCCCCTGTTCACACGTTTTACGGCCGCCGCCGCGAGGGAGCTCGGTGTTCCCGTTCCCGATGTTTGCCCACTGCTGCACAAAGTGCTGCTGGGCCACGACTGGCCCGGCAATATCCGTGAGCTCAAGGCGGCAGCCAAACGCCATGTGCTGGGTTTCCCCTTGCTGGGCGCCGAGCCGCAGGGCGAAGAGCACTTGGCCTGTGGGCTCAAATCGCAATTGCGAGTGATCGAAAAAGCCCTGATTCAGGAGTCGCTCAAGCGCCACGACAATTGTGTGGATTCGGTAAGCCTGGAACTGGACGTGCCACGCCGTACGCTCTATCGACGCATCAAAGAATTGCAGATCTAATAACCAGGCATCAAATAAAACGAAAGGCTCAGTCGAAAGACTGGGCCTTTCGTTTTATCTGTTGTTTGTCGGTGAACGCTCTCTACTAGAGTCACACTGGCTCACCTTCGGGTGGGCCTTTCTGCGTTTATATAGACCTTTACGCCGCTGGAGCAGGAATGCGGTGAGCATCACATCACCACAATTCAGCAAATTGTGAACATCATCACGTTCATCTTTCCCTGGTTGCCAATGGCCCATTTTCCTGTCAGTAACGAGAAGGTCGCGAATTGAGGCGCTTTTTAGACTGGTCGT**A**TACTAGAGTCACACAGGAAACCTACTAGATGAGTACAGGCATCGATAAGGACGTCCGAGAGTGTTGGGGCGTAACTGCATTATCAGCGGGTCATCAAATTGCAATGAATAGCGCGTTTCTGGATATGGACTTGCTGTTGTGCGGGGAAACCGGCACCGGCAAGGACACACTGGCCAACCGCATTCACGAGTTGTCCAGCAGGTCGGGACCCTTTGTGGGCATGAACTGCGCCGCCATTCCCGAGTCGCTGGCAGAGAGCCAGTTATTCGGTGTGGTCAACGGTGCATTCACCGGCGTATGCCGGGCTCGCGAGGGCTACATAGAGGCCTCCAGTGGTGGCACCTTGTACCTGGATGAAATCGACAGCATGCCGTTGAGCCTGCAAGCCAAACTGCTGCGTGTGTTGGAGAGTCGAGGTATCGAGCGTCTGGGCTCGACCGAATTTATCCCGGTGGATCTGCGGATCATTGCCTCGGCCCAGCGGCCACTGGATGAACTGGTGGAACAAGGACTTTTCCGTCGCGACCTGTTTTTTCGGCTCAACGTGCTGACGCTTCACTTGCCAGCCTTGCGCAAACGTCGTGAACAGATCCTGCCATTGTTCGACCAGTTCACCCAGGGTATCGCTGCCGAGTTCGGACGTCCCGCTCCTGCGCTGGACAGCGGGCGTGTGCAGCTGCTGCTCAGCCACGACTGGCCGGGCAACATCCGCGAATTGAAGTCTGCGGCCAAGCGCTTCGTACTCGGCTTCCCCTTGCTGGGCGCCGACCCTGTGGAAGCGCTTGACCCTGCCACGGGGCTGCGCACGCAAATGCGCATCATCGAGAAAATGCTCATCCAGGATGCCTTGAAGCGGCACAGGCACAATTTCGACGCGGTGCTTCAGGAGTTGGAGTTGCCAAGACGCACCCTGTATCACCGCATGAAGGAACTGGGAGTTGCAGCGCCGATCGCTGCGACGGCCGGGGTCTAATAACCAGGCATCAAATAAAACGAAAGGCTCAGTCGAAAGACTGGGCCTTTCGTTTTATCTGTTGTTTGTCGGTGAACGCTCTCTACTAGAGTCACACTGGCTCACCTTCGGGTGGGCCTTTCTGCGTTTATATACTAGAtttacagctagctcagtcctaggtattatgctagcTACTAGAGGGAGCCCAAATGACCGTAttacatagtgtggatttttttccgtctggtaacgcgtccgtggcgatagaaccccggctcccgcaggcggattttcctgaacatcatcatgattttcatgaaattgtgattgtcgaacatggcacgggtattcatgtgtttaatgggcagccctataccatcaccggtggcacggtctgtttcgtacgcgatcatgatcggcatctgtatgaacataccgataatctgtgtctgaccaatgtgctgtatcgctcgccggatcgatttcagtttctcgccgggctgaatcagttgctgccacaagagctggatgggcagtatccgtctcactggcgcgttaaccacagcgtattgcagcaggtgcgacagctggttgcacagatggaacagcaggaaggggaaaatgatttaccctcgaccgccagtcgcgagatcttgtttatgcaattactgctcttgctgcgtaaaagcagtttgcaggagaacctggaaaacagcgcatcacgtctcaacttgcttctggcctggctggaggaccattttgccgatgaggtgaattgggatgccgtggcggatcaattttctctttcactgcgtacgctacatcggcagcttaagcagcaaacgggactgacgcctcagcgatacctgaaccgcctgcgactgatgaaagcccgacatctgctacgccacagcgaggccagcgttactgacatcgcctatcgctgtggattcagcgacagtaaccacttttcgacgctttttcgccgagagtttaactggtcaccgcgtgatattcgccagggacgggatggctttctgcaataaCGAGAGAGAGTTAACatggctgaagcgcaaaatgatcccctgctgccgggatactcgtttaacgcccatctggtggcgggtttaacgccgattgaggccaacggttatctcgatttttttatcgaccgaccgctgggaatgaaaggttatattctcaatctcaccattcgcggtcagggggtggtgaaaaatcagggacgagaatttgtctgccgaccgggtgatattttgctgttcccgccaggagagattcatcactacggtcgtcatccggaggctcgcgaatggtatcaccagtgggtttactttcgtccgcgcgcctactggcatgaatggcttaactggccgtcaatatttgccaatacgggtttctttcgcccggatgaagcgcaccagccgcatttcagcgacctgtttgggcaaatcattaacgccgggcaaggggaagggcgctattcggagctgctggcgataaatctgcttgagcaattgttactgcggcgcatggaagcgattaacgagtcgctccatccaccgatggataatcgggtacgcgaggcttgtcagtacatcagcgatcacctggcagacagcaattttgatatcgccagcgtcgcacagcatgtttgcttgtcgccgtcgcgtctgtcacatcttttccgccagcagttagggattagcgtcttaagctggcgcgaggaccaacgcattagtcaggcgaagctgcttttgagcactacccggatgcctatcgccaccgtcggtcgcaatgttggttttgacgatcaactctatttctcgcgagtatttaaaaaatgcaccggggccagcccgagcgagtttcgtgccggttgtgaagaaaaagtgaatgatgtagccgtcaagttgtcataaccaggcatcaaataaaacgaaaggctcagtcgaaagactgggcctttcgttttatctgttgtttgtcggtgaacgctctctactagagtcacactggctcaccttcgggtgggcctttctgcgtttata

**XOR GATE I**

pHrpL.Rbs.λcl.T15-pBAD.pRHAB_Cl2B_.RbsB.RFP_asv_.T15 [pSB1C3]

GCCGGATTATGTCCGCTGAGTGGGTCACGGTCCCGGATCAGTTCCCTTGCGAAGCTGACCGATGTTTTTGTGCCAAAAGCTGTTGTGGCAAAAAACGGTTTGCGCAAAGTTTTGTATTACAAAGAATTTCACATTTTAAAATATCTTTATAAATCAATCAGTTATTTCTATTTTTAAGCTGGCATGGTTATCGCTATAGGGCTTGTACTACTAGAAAAGAGGAGAAATACTAGATGAGCACAAAAAAGAAACCATTAACACAAGAGCAGCTTGAGGACGCACGTCGCCTTAAAGCAATTTATGAAAAAAAGAAAAATGAACTTGGCTTATCCCAGGAATCTGTCGCAGACAAGATGGGGATGGGGCAGTCAGGCGTTGGTGCTTTATTTAATGGCATCAATGCATTAAATGCTTATAACGCCGCATTGCTTGCAAAAATTCTCAAAGTTAGCGTTGAAGAATTTAGCCCTTCAATCGCCAGAGAAATCTACGAGATGTATGAAGCGGTTAGTATGCAGCCGTCACTTAGAAGTGAGTATGAGTACCCTGTTTTTTCTCATGTTCAGGCAGGGATGTTCTCACCTGAGCTTAGAACCTTTACCAAAGGTGATGCGGAGAGATGGGTAAGCACAACCAAAAAAGCCAGTGATTCTGCATTCTGGCTTGAGGTTGAAGGTAATTCCATGACCGCACCAACAGGCTCCAAGCCAAGCTTTCCTGACGGAATGTTAATTCTCGTTGACCCTGAGCAGGCTGTTGAGCCAGGTGATTTCTGCATAGCCAGACTTGGGGGTGATGAGTTTACCTTCAAGAAACTGATCAGGGATAGCGGTCAGGTGTTTTTACAACCACTAAACCCACAGTACCCAATGATCCCATGCAATGAGAGTTGTTCCGTTGTGGGGAAAGTTATCGCTAGTCAGTGGCCTGAAGAGACGTTTGGCTAATAATACTAGAGCCAGGCATCAAATAAAACGAAAGGCTCAGTCGAAAGACTGGGCCTTTCGTTTTATCTGTTGTTTGTCGGTGAACGCTCTCTACTAGAGTCACACTGGCTCACCTTCGGGTGGGCCTTTCTGCGTTTATATACTAGAAGAAGAAACCAATTGTCCATATTGCATCAGACATTGCCGTCACTGCGTCTTTTACTGGCTCTTCTCGCTAACCAAACCGGTAACCCCGCTTATTAAAAGCATTCTGTAACAAAGCGGGACCAAAGCCATGACAAAAACGCGTAACAAAAGTGTCTATAATCACGGCAGAAAAGTCCACATTGATTATTTGCACGGCGTCACACTTTGCTATGCCATAGCATTTTTATCCATAAGATTAGCGGATTCTACCTGACGCTTTTTATCGCAACTCTCTACTGTTTCTCCATATACTAGACAGGAATGCGGTGAGCATCACATCACCACAATTCAGCAAATTGTGAACATCATCACGTTCATCTTTCCCTGGTTGCCAATGGCCCATTTTCCTGTCAGTAACGAGAAGGTCGCGAATTGAGGCGCTTTTTAGACTGGTCGTATATCACCGGGCGTGCTGATACAGTCATACTAGATCACACAGGAAAGTACTAGATGGCTTCCTCCGAAGACGTTATCAAAGAGTTCATGCGTTTCAAAGTTCGTATGGAAGGTTCCGTTAACGGTCACGAGTTCGAAATCGAAGGTGAAGGTGAAGGTCGTCCGTACGAAGGTACCCAGACCGCTAAACTGAAAGTTACCAAAGGTGGTCCGCTGCCGTTCGCTTGGGACATCCTGTCCCCGCAGTTCCAGTACGGTTCCAAAGCTTACGTTAAACACCCGGCTGACATCCCGGACTACCTGAAACTGTCCTTCCCGGAAGGTTTCAAATGGGAACGTGTTATGAACTTCGAAGACGGTGGTGTTGTTACCGTTACCCAGGACTCCTCCCTGCAAGACGGTGAGTTCATCTACAAAGTTAAACTGCGTGGTACCAACTTCCCGTCCGACGGTCCGGTTATGCAGAAAAAAACCATGGGTTGGGAAGCTTCCACCGAACGTATGTACCCGGAAGACGGTGCTCTGAAAGGTGAAATCAAAATGCGTCTGAAACTGAAAGACGGTGGTCACTACGACGCTGAAGTTAAAACCACCTACATGGCTAAAAAACCGGTTCAGCTGCCGGGTGCTTACAAAACCGACATCAAACTGGACATCACCTCCCACAACGAAGACTACACCATCGTTGAACAGTACGAACGTGCTGAAGGTCGTCACTCCACCGGTGCTGCTGCAAACGACGAAAACTACGCTGCATCTGTATAATACTAGAGCCAGGCATCAAATAAAACGAAAGGCTCAGTCGAAAGACTGGGCCTTTCGTTTTATCTGTTGTTTGTCGGTGAACGCTCTCTACTAGAGTCACACTGGCTCACCTTCGGGTGGGCCTTTCTGCGTTTATA

**XOR GATE II**

pHrpL.Rbs.λcl.T15-pBAD_Cl2B_.pRHAB_Cl2B_.RbsB.RFP_asv_.T15 [pSB1C3]

GCCGGATTATGTCCGCTGAGTGGGTCACGGTCCCGGATCAGTTCCCTTGCGAAGCTGACCGATGTTTTTGTGCCAAAAGCTGTTGTGGCAAAAAACGGTTTGCGCAAAGTTTTGTATTACAAAGAATTTCACATTTTAAAATATCTTTATAAATCAATCAGTTATTTCTATTTTTAAGCTGGCATGGTTATCGCTATAGGGCTTGTACTACTAGAAAAGAGGAGAAATACTAGATGAGCACAAAAAAGAAACCATTAACACAAGAGCAGCTTGAGGACGCACGTCGCCTTAAAGCAATTTATGAAAAAAAGAAAAATGAACTTGGCTTATCCCAGGAATCTGTCGCAGACAAGATGGGGATGGGGCAGTCAGGCGTTGGTGCTTTATTTAATGGCATCAATGCATTAAATGCTTATAACGCCGCATTGCTTGCAAAAATTCTCAAAGTTAGCGTTGAAGAATTTAGCCCTTCAATCGCCAGAGAAATCTACGAGATGTATGAAGCGGTTAGTATGCAGCCGTCACTTAGAAGTGAGTATGAGTACCCTGTTTTTTCTCATGTTCAGGCAGGGATGTTCTCACCTGAGCTTAGAACCTTTACCAAAGGTGATGCGGAGAGATGGGTAAGCACAACCAAAAAAGCCAGTGATTCTGCATTCTGGCTTGAGGTTGAAGGTAATTCCATGACCGCACCAACAGGCTCCAAGCCAAGCTTTCCTGACGGAATGTTAATTCTCGTTGACCCTGAGCAGGCTGTTGAGCCAGGTGATTTCTGCATAGCCAGACTTGGGGGTGATGAGTTTACCTTCAAGAAACTGATCAGGGATAGCGGTCAGGTGTTTTTACAACCACTAAACCCACAGTACCCAATGATCCCATGCAATGAGAGTTGTTCCGTTGTGGGGAAAGTTATCGCTAGTCAGTGGCCTGAAGAGACGTTTGGCTAATAATACTAGAGCCAGGCATCAAATAAAACGAAAGGCTCAGTCGAAAGACTGGGCCTTTCGTTTTATCTGTTGTTTGTCGGTGAACGCTCTCTACTAGAGTCACACTGGCTCACCTTCGGGTGGGCCTTTCTGCGTTTATATACTAGAAGAAGAAACCAATTGTCCATATTGCATCAGACATTGCCGTCACTGCGTCTTTTACTGGCTCTTCTCGCTAACCAAACCGGTAACCCCGCTTATTAAAAGCATTCTGTAACAAAGCGGGACCAAAGCCATGACAAAAACGCGTAACAAAAGTGTCTATAATCACGGCAGAAAAGTCCACATTGATTATTTGCACGGCGTCACACTTTGCTATGCCATAGCATTTTTATCCATAAGATTAGCGGATTCTACCTGACGCTTTTTATCGCAACTCTCTACTGTTTCTCCATATATCACCGGGCGTGCTGATATACTAGACAGGAATGCGGTGAGCATCACATCACCACAATTCAGCAAATTGTGAACATCATCACGTTCATCTTTCCCTGGTTGCCAATGGCCCATTTTCCTGTCAGTAACGAGAAGGTCGCGAATTGAGGCGCTTTTTAGACTGGTCGTATATCACCGGGCGTGCTGATACAGTCATACTAGATCACACAGGAAAGTACTAGATGGCTTCCTCCGAAGACGTTATCAAAGAGTTCATGCGTTTCAAAGTTCGTATGGAAGGTTCCGTTAACGGTCACGAGTTCGAAATCGAAGGTGAAGGTGAAGGTCGTCCGTACGAAGGTACCCAGACCGCTAAACTGAAAGTTACCAAAGGTGGTCCGCTGCCGTTCGCTTGGGACATCCTGTCCCCGCAGTTCCAGTACGGTTCCAAAGCTTACGTTAAACACCCGGCTGACATCCCGGACTACCTGAAACTGTCCTTCCCGGAAGGTTTCAAATGGGAACGTGTTATGAACTTCGAAGACGGTGGTGTTGTTACCGTTACCCAGGACTCCTCCCTGCAAGACGGTGAGTTCATCTACAAAGTTAAACTGCGTGGTACCAACTTCCCGTCCGACGGTCCGGTTATGCAGAAAAAAACCATGGGTTGGGAAGCTTCCACCGAACGTATGTACCCGGAAGACGGTGCTCTGAAAGGTGAAATCAAAATGCGTCTGAAACTGAAAGACGGTGGTCACTACGACGCTGAAGTTAAAACCACCTACATGGCTAAAAAACCGGTTCAGCTGCCGGGTGCTTACAAAACCGACATCAAACTGGACATCACCTCCCACAACGAAGACTACACCATCGTTGAACAGTACGAACGTGCTGAAGGTCGTCACTCCACCGGTGCTGCTGCAAACGACGAAAACTACGCTGCATCTGTATAATACTAGAGCCAGGCATCAAATAAAACGAAAGGCTCAGTCGAAAGACTGGGCCTTTCGTTTTATCTGTTGTTTGTCGGTGAACGCTCTCTACTAGAGTCACACTGGCTCACCTTCGGGTGGGCCTTTCTGCGTTTATA

**XOR GATE II**

pHrpL.Rbs.λcl.T15-pBAD_Cl2B_.pRHAB_Cl2B_.RbsB.RFP.T15 [pSB1C3]

GCCGGATTATGTCCGCTGAGTGGGTCACGGTCCCGGATCAGTTCCCTTGCGAAGCTGACCGATGTTTTTGTGCCAAAAGCTGTTGTGGCAAAAAACGGTTTGCGCAAAGTTTTGTATTACAAAGAATTTCACATTTTAAAATATCTTTATAAATCAATCAGTTATTTCTATTTTTAAGCTGGCATGGTTATCGCTATAGGGCTTGTACTACTAGAAAAGAGGAGAAATACTAGATGAGCACAAAAAAGAAACCATTAACACAAGAGCAGCTTGAGGACGCACGTCGCCTTAAAGCAATTTATGAAAAAAAGAAAAATGAACTTGGCTTATCCCAGGAATCTGTCGCAGACAAGATGGGGATGGGGCAGTCAGGCGTTGGTGCTTTATTTAATGGCATCAATGCATTAAATGCTTATAACGCCGCATTGCTTGCAAAAATTCTCAAAGTTAGCGTTGAAGAATTTAGCCCTTCAATCGCCAGAGAAATCTACGAGATGTATGAAGCGGTTAGTATGCAGCCGTCACTTAGAAGTGAGTATGAGTACCCTGTTTTTTCTCATGTTCAGGCAGGGATGTTCTCACCTGAGCTTAGAACCTTTACCAAAGGTGATGCGGAGAGATGGGTAAGCACAACCAAAAAAGCCAGTGATTCTGCATTCTGGCTTGAGGTTGAAGGTAATTCCATGACCGCACCAACAGGCTCCAAGCCAAGCTTTCCTGACGGAATGTTAATTCTCGTTGACCCTGAGCAGGCTGTTGAGCCAGGTGATTTCTGCATAGCCAGACTTGGGGGTGATGAGTTTACCTTCAAGAAACTGATCAGGGATAGCGGTCAGGTGTTTTTACAACCACTAAACCCACAGTACCCAATGATCCCATGCAATGAGAGTTGTTCCGTTGTGGGGAAAGTTATCGCTAGTCAGTGGCCTGAAGAGACGTTTGGCTAATAATACTAGAGCCAGGCATCAAATAAAACGAAAGGCTCAGTCGAAAGACTGGGCCTTTCGTTTTATCTGTTGTTTGTCGGTGAACGCTCTCTACTAGAGTCACACTGGCTCACCTTCGGGTGGGCCTTTCTGCGTTTATATACTAGAAGAAGAAACCAATTGTCCATATTGCATCAGACATTGCCGTCACTGCGTCTTTTACTGGCTCTTCTCGCTAACCAAACCGGTAACCCCGCTTATTAAAAGCATTCTGTAACAAAGCGGGACCAAAGCCATGACAAAAACGCGTAACAAAAGTGTCTATAATCACGGCAGAAAAGTCCACATTGATTATTTGCACGGCGTCACACTTTGCTATGCCATAGCATTTTTATCCATAAGATTAGCGGATTCTACCTGACGCTTTTTATCGCAACTCTCTACTGTTTCTCCATATATCACCGGGCGTGCTGATATACTAGACAGGAATGCGGTGAGCATCACATCACCACAATTCAGCAAATTGTGAACATCATCACGTTCATCTTTCCCTGGTTGCCAATGGCCCATTTTCCTGTCAGTAACGAGAAGGTCGCGAATTGAGGCGCTTTTTAGACTGGTCGTATATCACCGGGCGTGCTGATACAGTCATACTAGATCACACAGGAAAGTACTAGATGGCTTCCTCCGAAGACGTTATCAAAGAGTTCATGCGTTTCAAAGTTCGTATGGAAGGTTCCGTTAACGGTCACGAGTTCGAAATCGAAGGTGAAGGTGAAGGTCGTCCGTACGAAGGTACCCAGACCGCTAAACTGAAAGTTACCAAAGGTGGTCCGCTGCCGTTCGCTTGGGACATCCTGTCCCCGCAGTTCCAGTACGGTTCCAAAGCTTACGTTAAACACCCGGCTGACATCCCGGACTACCTGAAACTGTCCTTCCCGGAAGGTTTCAAATGGGAACGTGTTATGAACTTCGAAGACGGTGGTGTTGTTACCGTTACCCAGGACTCCTCCCTGCAAGACGGTGAGTTCATCTACAAAGTTAAACTGCGTGGTACCAACTTCCCGTCCGACGGTCCGGTTATGCAGAAAAAAACCATGGGTTGGGAAGCTTCCACCGAACGTATGTACCCGGAAGACGGTGCTCTGAAAGGTGAAATCAAAATGCGTCTGAAACTGAAAGACGGTGGTCACTACGACGCTGAAGTTAAAACCACCTACATGGCTAAAAAACCGGTTCAGCTGCCGGGTGCTTACAAAACCGACATCAAACTGGACATCACCTCCCACAACGAAGACTACACCATCGTTGAACAGTACGAACGTGCTGAAGGTCGTCACTCCACCGGTGCTTAATACTAGAGCCAGGCATCAAATAAAACGAAAGGCTCAGTCGAAAGACTGGGCCTTTCGTTTTATCTGTTGTTTGTCGGTGAACGCTCTCTACTAGAGTCACACTGGCTCACCTTCGGGTGGGCCTTTCTGCGTTTATA

**XOR GATE IV**

pHrpL.Rbs.λcl.T15-pBAD_Cl2B_.RbsB.RFP_asv_.T15-pRHAB_Cl2B_.RbsB.RFP_asv_.T15 [pSB1C3]

GCCGGATTATGTCCGCTGAGTGGGTCACGGTCCCGGATCAGTTCCCTTGCGAAGCTGACCGATGTTTTTGTGCCAAAAGCTGTTGTGGCAAAAAACGGTTTGCGCAAAGTTTTGTATTACAAAGAATTTCACATTTTAAAATATCTTTATAAATCAATCAGTTATTTCTATTTTTAAGCTGGCATGGTTATCGCTATAGGGCTTGTACTACTAGAAAAGAGGAGAAATACTAGATGAGCACAAAAAAGAAACCATTAACACAAGAGCAGCTTGAGGACGCACGTCGCCTTAAAGCAATTTATGAAAAAAAGAAAAATGAACTTGGCTTATCCCAGGAATCTGTCGCAGACAAGATGGGGATGGGGCAGTCAGGCGTTGGTGCTTTATTTAATGGCATCAATGCATTAAATGCTTATAACGCCGCATTGCTTGCAAAAATTCTCAAAGTTAGCGTTGAAGAATTTAGCCCTTCAATCGCCAGAGAAATCTACGAGATGTATGAAGCGGTTAGTATGCAGCCGTCACTTAGAAGTGAGTATGAGTACCCTGTTTTTTCTCATGTTCAGGCAGGGATGTTCTCACCTGAGCTTAGAACCTTTACCAAAGGTGATGCGGAGAGATGGGTAAGCACAACCAAAAAAGCCAGTGATTCTGCATTCTGGCTTGAGGTTGAAGGTAATTCCATGACCGCACCAACAGGCTCCAAGCCAAGCTTTCCTGACGGAATGTTAATTCTCGTTGACCCTGAGCAGGCTGTTGAGCCAGGTGATTTCTGCATAGCCAGACTTGGGGGTGATGAGTTTACCTTCAAGAAACTGATCAGGGATAGCGGTCAGGTGTTTTTACAACCACTAAACCCACAGTACCCAATGATCCCATGCAATGAGAGTTGTTCCGTTGTGGGGAAAGTTATCGCTAGTCAGTGGCCTGAAGAGACGTTTGGCTAATAATACTAGAGCCAGGCATCAAATAAAACGAAAGGCTCAGTCGAAAGACTGGGCCTTTCGTTTTATCTGTTGTTTGTCGGTGAACGCTCTCTACTAGAGTCACACTGGCTCACCTTCGGGTGGGCCTTTCTGCGTTTATATACTAGAAGAAGAAACCAATTGTCCATATTGCATCAGACATTGCCGTCACTGCGTCTTTTACTGGCTCTTCTCGCTAACCAAACCGGTAACCCCGCTTATTAAAAGCATTCTGTAACAAAGCGGGACCAAAGCCATGACAAAAACGCGTAACAAAAGTGTCTATAATCACGGCAGAAAAGTCCACATTGATTATTTGCACGGCGTCACACTTTGCTATGCCATAGCATTTTTATCCATAAGATTAGCGGATTCTACCTGACGCTTTTTATCGCAACTCTCTACTGTTTCTCCATATATCACCGGGCGTGCTGATACAGTCATACTAGATCACACAGGAAAGTACTAATGGCTTCCTCCGAAGACGTTATCAAAGAGTTCATGCGTTTCAAAGTTCGTATGGAAGGTTCCGTTAACGGTCACGAGTTCGAAATCGAAGGTGAAGGTGAAGGTCGTCCGTACGAAGGTACCCAGACCGCTAAACTGAAAGTTACCAAAGGTGGTCCGCTGCCGTTCGCTTGGGACATCCTGTCCCCGCAGTTCCAGTACGGTTCCAAAGCTTACGTTAAACACCCGGCTGACATCCCGGACTACCTGAAACTGTCCTTCCCGGAAGGTTTCAAATGGGAACGTGTTATGAACTTCGAAGACGGTGGTGTTGTTACCGTTACCCAGGACTCCTCCCTGCAAGACGGTGAGTTCATCTACAAAGTTAAACTGCGTGGTACCAACTTCCCGTCCGACGGTCCGGTTATGCAGAAAAAAACCATGGGTTGGGAAGCTTCCACCGAACGTATGTACCCGGAAGACGGTGCTCTGAAAGGTGAAATCAAAATGCGTCTGAAACTGAAAGACGGTGGTCACTACGACGCTGAAGTTAAAACCACCTACATGGCTAAAAAACCGGTTCAGCTGCCGGGTGCTTACAAAACCGACATCAAACTGGACATCACCTCCCACAACGAAGACTACACCATCGTTGAACAGTACGAACGTGCTGAAGGTCGTCACTCCACCGGTGCTGCTGCAAACGACGAAAACTACGCTGCATCTGTATAATACTAGAGCCAGGCATCAAATAAAACGAAAGGCTCAGTCGAAAGACTGGGCCTTTCGTTTTATCTGTTGTTTGTCGGTGAACGCTCTCTACTAGAGTCACACTGGCTCACCTTCGGGTGGGCCTTTCTGCGTTTATATACTAGACAGGAATGCGGTGAGCATCACATCACCACAATTCAGCAAATTGTGAACATCATCACGTTCATCTTTCCCTGGTTGCCAATGGCCCATTTTCCTGTCAGTAACGAGAAGGTCGCGAATTGAGGCGCTTTTTAGACTGGTCGTATATCACCGGGCGTGCTGATACAGTCATACTAGATCACACAGGAAAGTACTAGATGGCTTCCTCCGAAGACGTTATCAAAGAGTTCATGCGTTTCAAAGTTCGTATGGAAGGTTCCGTTAACGGTCACGAGTTCGAAATCGAAGGTGAAGGTGAAGGTCGTCCGTACGAAGGTACCCAGACCGCTAAACTGAAAGTTACCAAAGGTGGTCCGCTGCCGTTCGCTTGGGACATCCTGTCCCCGCAGTTCCAGTACGGTTCCAAAGCTTACGTTAAACACCCGGCTGACATCCCGGACTACCTGAAACTGTCCTTCCCGGAAGGTTTCAAATGGGAACGTGTTATGAACTTCGAAGACGGTGGTGTTGTTACCGTTACCCAGGACTCCTCCCTGCAAGACGGTGAGTTCATCTACAAAGTTAAACTGCGTGGTACCAACTTCCCGTCCGACGGTCCGGTTATGCAGAAAAAAACCATGGGTTGGGAAGCTTCCACCGAACGTATGTACCCGGAAGACGGTGCTCTGAAAGGTGAAATCAAAATGCGTCTGAAACTGAAAGACGGTGGTCACTACGACGCTGAAGTTAAAACCACCTACATGGCTAAAAAACCGGTTCAGCTGCCGGGTGCTTACAAAACCGACATCAAACTGGACATCACCTCCCACAACGAAGACTACACCATCGTTGAACAGTACGAACGTGCTGAAGGTCGTCACTCCACCGGTGCTGCTGCAAACGACGAAAACTACGCTGCATCTGTATAATACTAGAGCCAGGCATCAAATAAAACGAAAGGCTCAGTCGAAAGACTGGGCCTTTCGTTTTATCTGTTGTTTGTCGGTGAACGCTCTCTACTAGAGTCACACTGGCTCACCTTCGGGTGGGCCTTTCTGCGTTTATA

**HALF ADDER**

The HALF ADDER was created using DNA sequences of AND GATE and XOR GATE IV as described above.

**HALF SUBTRACTOR**

The HALF SUBTRACTOR was created using DNA sequences of plasmid pAW-SUB and XOR GATE IV.

pBAD.Rbs.HrpS.T15-pRHAB.Rbs.HrpR.T15-pCON.Rbs.RhaS.Rbs.AraC.T15-pBAD_Cl2B_.Rbs.GFP.T15 [Designated pAW-SUB, pSB4A5]

agaagaaaccaattgtccatattgcatcagacattgccgtcactgcgtcttttactggctcttctcgctaaccaaaccggtaaccccgcttattaaaagcattctgtaacaaagcgggaccaaagccatgacaaaaacgcgtaacaaaagtgtctataatcacggcagaaaagtccacattgattatttgcacggcgtcacactttgctatgccatagcatttttatccataagattagcggattctacctgacgctttttatcgcaactctctactgtttctccaT**A**TACTAGAGTCACACAGGACTACTAGATGACTATAATGATGAGTCTTGATGAAAGGTTTGAGGATGATCTGGACGAGGAGCGGGTTCCGAATCTGGGGATAGTTGCCGAAAGTATTTCGCAACTGGGTATCGACGTGCTGCTATCGGGTGAGACCGGCACGGGCAAAGACACGATTGCCCGACGGATTCATGAGATGTCAGGCCGCAAAGGGCGCCTGGTGGCGATGAATTGCGCGGCCATTCCGGAGTCCCTCGCCGAGAGCGAGTTATTCGGCGTGGTCAGCGGTGCCTACACCGGCGCTGATCGCTCCAGAGTCGGTTATGTCGAAGCGGCGCAGGGCGGCACGCTGTACCTGGATGAGATCGATAGCATGCCGCTGAGCCTGCAAGCCAAATTGCTGAGGGTGCTGGAAACCCGAGCGCTTGAACGGCTGGGTTCGACGTCGACGATCAAGCTGGATATCTGCGTGATCGCCTCCGCCCAATGCTCGCTGGACGACGCCGTCGAGCGGGGGCAGTTTCGTCGCGATCTGTATTTTCGCCTGAACGTCCTGACACTCAAGCTTCCTCCGCTACGTAACCAGTCTGATCGCATAGTTCCCCTGTTCACACGTTTTACGGCCGCCGCCGCGAGGGAGCTCGGTGTTCCCGTTCCCGATGTTTGCCCACTGCTGCACAAAGTGCTGCTGGGCCACGACTGGCCCGGCAATATCCGTGAGCTCAAGGCGGCAGCCAAACGCCATGTGCTGGGTTTCCCCTTGCTGGGCGCCGAGCCGCAGGGCGAAGAGCACTTGGCCTGTGGGCTCAAATCGCAATTGCGAGTGATCGAAAAAGCCCTGATTCAGGAGTCGCTCAAGCGCCACGACAATTGTGTGGATTCGGTAAGCCTGGAACTGGACGTGCCACGCCGTACGCTCTATCGACGCATCAAAGAATTGCAGATCTAATAACCAGGCATCAAATAAAACGAAAGGCTCAGTCGAAAGACTGGGCCTTTCGTTTTATCTGTTGTTTGTCGGTGAACGCTCTCTACTAGAGTCACACTGGCTCACCTTCGGGTGGGCCTTTCTGCGTTTATATAGACCTTTACGCCGCTGGAGCAGGAATGCGGTGAGCATCACATCACCACAATTCAGCAAATTGTGAACATCATCACGTTCATCTTTCCCTGGTTGCCAATGGCCCATTTTCCTGTCAGTAACGAGAAGGTCGCGAATTGAGGCGCTTTTTAGACTGGTCGT**A**TACTAGAGTCACACAGGAAACCTACTAGATGAGTACAGGCATCGATAAGGACGTCCGAGAGTGTTGGGGCGTAACTGCATTATCAGCGGGTCATCAAATTGCAATGAATAGCGCGTTTCTGGATATGGACTTGCTGTTGTGCGGGGAAACCGGCACCGGCAAGGACACACTGGCCAACCGCATTCACGAGTTGTCCAGCAGGTCGGGACCCTTTGTGGGCATGAACTGCGCCGCCATTCCCGAGTCGCTGGCAGAGAGCCAGTTATTCGGTGTGGTCAACGGTGCATTCACCGGCGTATGCCGGGCTCGCGAGGGCTACATAGAGGCCTCCAGTGGTGGCACCTTGTACCTGGATGAAATCGACAGCATGCCGTTGAGCCTGCAAGCCAAACTGCTGCGTGTGTTGGAGAGTCGAGGTATCGAGCGTCTGGGCTCGACCGAATTTATCCCGGTGGATCTGCGGATCATTGCCTCGGCCCAGCGGCCACTGGATGAACTGGTGGAACAAGGACTTTTCCGTCGCGACCTGTTTTTTCGGCTCAACGTGCTGACGCTTCACTTGCCAGCCTTGCGCAAACGTCGTGAACAGATCCTGCCATTGTTCGACCAGTTCACCCAGGGTATCGCTGCCGAGTTCGGACGTCCCGCTCCTGCGCTGGACAGCGGGCGTGTGCAGCTGCTGCTCAGCCACGACTGGCCGGGCAACATCCGCGAATTGAAGTCTGCGGCCAAGCGCTTCGTACTCGGCTTCCCCTTGCTGGGCGCCGACCCTGTGGAAGCGCTTGACCCTGCCACGGGGCTGCGCACGCAAATGCGCATCATCGAGAAAATGCTCATCCAGGATGCCTTGAAGCGGCACAGGCACAATTTCGACGCGGTGCTTCAGGAGTTGGAGTTGCCAAGACGCACCCTGTATCACCGCATGAAGGAACTGGGAGTTGCAGCGCCGATCGCTGCGACGGCCGGGGTCTAATAACCAGGCATCAAATAAAACGAAAGGCTCAGTCGAAAGACTGGGCCTTTCGTTTTATCTGTTGTTTGTCGGTGAACGCTCTCTACTAGAGTCACACTGGCTCACCTTCGGGTGGGCCTTTCTGCGTTTATATACTAGAtttacagctagctcagtcctaggtattatgctagcTACTAGAGGGAGCCCAAATGACCGTAttacatagtgtggatttttttccgtctggtaacgcgtccgtggcgatagaaccccggctcccgcaggcggattttcctgaacatcatcatgattttcatgaaattgtgattgtcgaacatggcacgggtattcatgtgtttaatgggcagccctataccatcaccggtggcacggtctgtttcgtacgcgatcatgatcggcatctgtatgaacataccgataatctgtgtctgaccaatgtgctgtatcgctcgccggatcgatttcagtttctcgccgggctgaatcagttgctgccacaagagctggatgggcagtatccgtctcactggcgcgttaaccacagcgtattgcagcaggtgcgacagctggttgcacagatggaacagcaggaaggggaaaatgatttaccctcgaccgccagtcgcgagatcttgtttatgcaattactgctcttgctgcgtaaaagcagtttgcaggagaacctggaaaacagcgcatcacgtctcaacttgcttctggcctggctggaggaccattttgccgatgaggtgaattgggatgccgtggcggatcaattttctctttcactgcgtacgctacatcggcagcttaagcagcaaacgggactgacgcctcagcgatacctgaaccgcctgcgactgatgaaagcccgacatctgctacgccacagcgaggccagcgttactgacatcgcctatcgctgtggattcagcgacagtaaccacttttcgacgctttttcgccgagagtttaactggtcaccgcgtgatattcgccagggacgggatggctttctgcaataaCGAGAGAGAGTTAACatggctgaagcgcaaaatgatcccctgctgccgggatactcgtttaacgcccatctggtggcgggtttaacgccgattgaggccaacggttatctcgatttttttatcgaccgaccgctgggaatgaaaggttatattctcaatctcaccattcgcggtcagggggtggtgaaaaatcagggacgagaatttgtctgccgaccgggtgatattttgctgttcccgccaggagagattcatcactacggtcgtcatccggaggctcgcgaatggtatcaccagtgggtttactttcgtccgcgcgcctactggcatgaatggcttaactggccgtcaatatttgccaatacgggtttctttcgcccggatgaagcgcaccagccgcatttcagcgacctgtttgggcaaatcattaacgccgggcaaggggaagggcgctattcggagctgctggcgataaatctgcttgagcaattgttactgcggcgcatggaagcgattaacgagtcgctccatccaccgatggataatcgggtacgcgaggcttgtcagtacatcagcgatcacctggcagacagcaattttgatatcgccagcgtcgcacagcatgtttgcttgtcgccgtcgcgtctgtcacatcttttccgccagcagttagggattagcgtcttaagctggcgcgaggaccaacgcattagtcaggcgaagctgcttttgagcactacccggatgcctatcgccaccgtcggtcgcaatgttggttttgacgatcaactctatttctcgcgagtatttaaaaaatgcaccggggccagcccgagcgagtttcgtgccggttgtgaagaaaaagtgaatgatgtagccgtcaagttgtcataaccaggcatcaaataaaacgaaaggctcagtcgaaagactgggcctttcgttttatctgttgtttgtcggtgaacgctctctactagagtcacactggctcaccttcgggtgggcctttctgcgtttataAGAAGAAACCAATTGTCCATATTGCATCAGACATTGCCGTCACTGCGTCTTTTACTGGCTCTTCTCGCTAACCAAACCGGTAACCCCGCTTATTAAAAGCATTCTGTAACAAAGCGGGACCAAAGCCATGACAAAAACGCGTAACAAAAGTGTCTATAATCACGGCAGAAAAGTCCACATTGATTATTTGCACGGCGTCACACTTTGCTATGCCATAGCATTTTTATCCATAAGATTAGCGGATTCTACCTGACGCTTTTTATCGCAACTCTCTACTGTTTCTCCATATATCACCGGGCGTGCTGATACAGTCATACTAGATTAAAGAGGAGAAATACTAGATGCGTAAAGGAGAAGAACTTTTCACTGGAGTTGTCCCAATTCTTGTTGAATTAGATGGTGATGTTAATGGGCACAAATTTTCTGTCAGTGGAGAGGGTGAAGGTGATGCAACATACGGAAAACTTACCCTTAAATTTATTTGCACTACTGGAAAACTACCTGTTCCATGGCCAACACTTGTCACTACTTTCGGTTATGGTGTTCAATGCTTTGCGAGATACCCAGATCATATGAAACAGCATGACTTTTTCAAGAGTGCCATGCCCGAAGGTTATGTACAGGAAAGAACTATATTTTTCAAAGATGACGGGAACTACAAGACACGTGCTGAAGTCAAGTTTGAAGGTGATACCCTTGTTAATAGAATCGAGTTAAAAGGTATTGATTTTAAAGAAGATGGAAACATTCTTGGACACAAATTGGAATACAACTATAACTCACACAATGTATACATCATGGCAGACAAACAAAAGAATGGAATCAAAGTTAACTTCAAAATTAGACACAACATTGAAGATGGAAGCGTTCAACTAGCAGACCATTATCAACAAAATACTCCAATTGGCGATGGCCCTGTCCTTTTACCAGACAACCATTACCTGTCCACACAATCTGCCCTTTCGAAAGATCCCAACGAAAAGAGAGACCACATGGTCCTTCTTGAGTTTGTAACAGCTGCTGGGATTACACATGGCATGGATGAACTATACAAATAATAATACTAGAGCCAGGCATCAAATAAAACGAAAGGCTCAGTCGAAAGACTGGGCCTTTCGTTTTATCTGTTGTTTGTCGGTGAACGCTCTCTACTAGAGTCACACTGGCTCACCTTCGGGTGGGCCTTTCTGCGTTTATA

**References**

1. Wang BJ, Kitney RI, Joly N, Buck M: **Engineering modular and orthogonal genetic logic gates for robust digital-like synthetic biology**. *Nature Communications* 2011, **2**.
